# Combined Impact of Smoking Duration and Malnutrition on Cancer Survival: Insights Into Systemic Inflammation

**DOI:** 10.1002/jcsm.70207

**Published:** 2026-02-17

**Authors:** Hong Zhao, Bing Yin, Zhe Zhao, Xiangrui Li, Changhong Xu, Yi Li, Zhaoting Bu, Xinyin Chen, Hanping Shi

**Affiliations:** ^1^ Department of Gastrointestinal Surgery/Department of Clinical Nutrition, Beijing Shijitan Hospital Capital Medical University Beijing China; ^2^ Key Laboratory of Cancer FSMP for State Market Regulation Beijing China; ^3^ Laboratory for Clinical Medicine Capital Medical University Beijing China; ^4^ Department of Obstetrics and Gynecology, Beijing Shijitan Hospital Capital Medical University Beijing China; ^5^ Department of Gastrointestinal and Gland Surgery, the First Affiliated Hospital Guangxi Medical University Nanning Guangxi China; ^6^ The First Affiliated Hospital of Wenzhou Medical University Wenzhou Medical University Wenzhou China

**Keywords:** cancer, inflammation, malnutrition, smoking, survival

## Abstract

**Background:**

Smoking and malnutrition are established risk factors for cancer survival, yet evidence on their combined effects remains limited. This study aimed to investigate the joint impact of smoking duration and malnutrition on overall survival (OS) in patients with solid tumours and to explore the potential underlying mechanism—systemic inflammation.

**Methods:**

This study was based on the Investigation of Nutrition Status and Clinical Outcomes in Common Cancers (INSCOC), which enrolled 29 988 patients with solid tumours and collected data on smoking status, nutritional status assessed by the Patient‐Generated Subjective Global Assessment (PG‐SGA) and haematological indicators. Restricted cubic spline regression, Cox proportional hazards models and logistic regression were used to examine the associations of smoking and malnutrition with OS and their relationships with inflammatory markers.

**Results:**

A total of 29 988 patients were included, among whom 7649 were smokers with malnutrition (male: 7095 [92.8%]; female: 554 [7.2%]); their mean age was 60.17 ± 10.57 years. Both smoking and malnutrition independently increased mortality risk in patients with solid tumours (HR = 1.24, 95% CI 1.15–1.35, *p* < 0.001; HR = 1.25, 95% CI 1.17–1.33, *p* < 0.001), with the highest risk observed in those with both risk factors (HR = 1.46, 95% CI 1.36–1.57, *p* < 0.001). Smoking duration showed a dose–response association with OS. Specifically, among smokers, every additional 5 years of smoking increased the risk of death by 6% (HR = 1.06, 95% CI 1.04–1.07, *p* < 0.001). In patients with malnutrition, every additional 10 years of smoking increased the risk of death by 86% (HR = 1.86, 95% CI 1.55–2.23, *p* < 0.001). Further analyses showed that, with increasing smoking duration, CRP increased from 3.50 [1.79, 13.60] to 6.00 [2.87, 24.90] mg/L and WBC from 5.70 [4.47, 7.31] to 6.47 [5.10, 8.40] × 10^9^/L (both *p* < 0.001). With worsening malnutrition, CRP rose from 3.11 [1.23, 6.28] to 10.00 [3.20, 40.70] mg/L and WBC from 5.70 [4.51, 7.24] to 6.39 [4.84, 8.71] × 10^9^/L (both *p* < 0.001), indicating that prolonged smoking and aggravated malnutrition were associated with elevated systemic inflammation.

**Conclusions:**

Smoking and malnutrition are crucial prognostic factors for survival in cancer patients. The results emphasize the critical role of managing smoking duration and improving nutritional status in the care of cancer patients. Clinically, attention should be given to controlling inflammation levels in patients with smoking and malnutrition.

## 1 Introduction

Cancer is the second leading cause of death globally [1]. In the United States, over 4 million cancer deaths have been avoided since 1991 due to the reduction in smoking, early detection of certain cancers and improvements in adjuvant and metastatic treatment options [2]. In China, lung cancer has become the leading cause of cancer‐related deaths, with smoking being the most important factor contributing to cancer mortality [3]. Cancer is a large category of various malignant tumours, the causes of which, either in whole or in part, are due to factors that individuals and governments can control through their choices. These factors include smoking and alcohol consumption, obesity, low intake of fruits and vegetables, lack of physical exercise and sun exposure. Therefore, if more people avoid these factors, a significant portion of the impact of cancer can be alleviated [4]. Despite the continuous advancement of cancer treatment technologies, patient survival rates are still affected by various complications, with smoking being a prominent modifiable independent risk factor [5, 6].

Smoking is a widely prevalent and highly harmful habit. The smoke produced by burning tobacco contains various harmful substances, including nicotine, tar and carbon monoxide [7]. Smoking reduces the effectiveness of most widely used cancer treatments, including immunotherapy, radiation therapy and chemotherapy [8]. Gemine et al.'s research found that smoking cessation was independently and significantly associated with improved survival in non–small cell lung cancer, regardless of its stage [9]. Among smokers, complete smoking cessation is still the most effective way to prevent cancer and cardiovascular diseases [10].

The causes of cancer‐related malnutrition are complex and are caused by malignant tumours that induce a systemic pro‐inflammatory state [11]. Malnutrition is associated with worse clinical outcomes in cancer patients [12]. Smoking is a risk factor for nutritional status in cancer patients [13]. On the one hand, smoking causes changes in taste and smell, reducing patients' appetite and food intake [14]. On the other hand, smoking is linked to a higher risk of sarcopenia [15].

Tumour‐associated inflammation is a characteristic of nearly all cancers, and inflammation is a recognized hallmark of cancer, significantly contributing to the development and progression of malignant tumours [16, 17]. Studies further show that the tumour microenvironment, mainly coordinated by inflammatory cells, plays an essential role in tumour progression, promoting proliferation, survival and migration. Inflammatory mediators and cellular effectors are vital components of the tumour's local environment [18, 19]. Smoking‐related oxidative stress induces inflammation, leading to further production of reactive oxygen species, which may increase oxidative damage to macromolecular targets, thus contributing to the initiation and/or progression of cancer [20]. Smoking can damage immune cell function and provoke inflammatory reactions, thereby promoting cancer progression [21].

Notably, smoking and malnutrition do not exist in isolation but may interact with and reinforce each other as risk factors. Smoking not only causes direct damage to the body through carcinogenic substances but also suppresses appetite, alters taste and smell and induces oxidative stress, inflammation and metabolic disorders [22], thereby potentially increasing the risk of malnutrition. A previous study demonstrated that paternal smoking was associated with an increased risk of malnutrition in children [23]. Smoking history has been identified as a significant risk factor associated with malnutrition in patients with stroke [24]. C‐reactive protein (CRP) is an important acute‐phase protein that reflects systemic inflammation, and its elevation is commonly observed in cancer patients with systemic inflammatory states [25]. CRP levels were significantly higher in malnourished patients compared with well‐nourished patients, indicating enhanced systemic inflammation in the malnourished group [26]. Malnutrition significantly reduces antioxidant levels and impairs T‐cell activity and NF‐κB regulatory function, thereby rendering the body more susceptible to oxidative stress [27]. Both factors converge through a shared pathological mechanism involving inflammation, leading to elevated pro‐inflammatory cytokines, increased oxidative damage and enhanced immunosuppression, thereby jointly promoting tumourigenesis and progression and shortening patient survival. However, most previous studies have examined smoking or malnutrition separately, and evidence regarding their potential synergistic effects via inflammatory pathways remains limited. Whether their coexistence exerts a stronger detrimental impact on cancer survival is still unclear. Therefore, this multicentre prospective cohort study aimed to evaluate whether smoking and malnutrition act in concert at the inflammatory level and whether their combined effect is more detrimental to patient outcomes than either factor alone.

## 2 Methods

### 2.1 Research Design and Participants

This study was based on the Investigation of Nutrition Status and Clinical Outcomes in Common Cancers (INSCOC), a multicentre prospective cohort study conducted in China since 2013. The study aimed to systematically collect clinical, nutritional and follow‐up data from cancer patients to evaluate the impact of nutritional status on clinical outcomes. The detailed study design and methodology have been previously published [28]. Inclusion criteria comprised patients aged ≥ 18 years with a pathological diagnosis of breast cancer, biliary tract carcinoma, bladder carcinoma, cervical cancer, colorectal cancer, endometrial cancer, oesophageal cancer, gastric cancer, liver cancer, lung cancer, malignant brain tumours, nasopharyngeal carcinoma, ovarian cancer, pancreatic cancer or prostate cancer. Exclusion criteria included severe infection, immunodeficiency, concurrent malignancies, incomplete serological data, serious cardiopulmonary comorbidities and lack of follow‐up. For individuals who were admitted multiple times during the study period, only data from the first hospitalization were collected. Written informed consent was obtained from all participants. The study was conducted in accordance with the principles of the Declaration of Helsinki. All participating centres and institutions received approval from the relevant Ethics Committee (registration no.: ChiCTR1800020329; ethics approval number: Medical Research Ethics Review [2013] 82). The Ethics Committee overseeing the study was the Medical Ethics Committee of the First Affiliated Hospital of Sun Yat‐sen University.

### 2.2 Assessment of Demographic Characteristics and Haematological Measurements

Data collection began at baseline, within 48 h of hospital admission. Baseline assessments were carried out by trained investigators using specialized equipment. Current smokers were defined as individuals who had smoked more than 100 cigarettes in their lifetime and who reported smoking either daily or occasionally at the time of the survey [29]. The specific smoking history was obtained through self‐report or from the patient's relatives, including the average number of packs smoked per day and the duration of smoking in years. The Patient‐Generated Subjective Global Assessment (PG‐SGA) is a nutritional assessment method adapted by Ottery for cancer patients [30]. Malnutrition was defined as a PG‐SGA score of 4 or greater, and the PG‐SGA has been widely used in studies of malnutrition among cancer patients in China [13]. Based on nutritional assessment, PG‐SGA scores were further categorized into four groups: 0–1 as normal, 2–3 as mild malnutrition, 4–8 as moderate malnutrition and ≥ 9 as severe malnutrition [31]. Within 24 h of hospital admission, patients underwent routine laboratory tests, including platelet count, total lymphocyte count and levels of haemoglobin, albumin, total protein, creatinine, CRP and other relevant markers, all of which were analysed by the clinical laboratories of the participating hospitals.

### 2.3 Outcomes and Covariates

The endpoint of this study was overall survival (OS), which was defined as the time from cancer diagnosis to death or the last follow‐up (30 June 2022).

Covariates included sex, age, TNM, cancer types, surgery, chemotherapy, radiotherapy, occupation and BMI.

### 2.4 Statistical Analysis

All data, whether normally distributed or not, were presented as means with standard deviations (SDs) or medians with interquartile ranges (IQRs), and comparisons between groups were made using *t*‐tests, analysis of variance tests or Mann–Whitney *U* tests. Categorical variables were presented as number and percentage and were compared between groups using the χ2 test. A restricted cubic spline regression model was used to analyse the relationship between PG‐SGA, smoking (packs, duration in years, pack‐years) and OS in cancer patients. Cox proportional hazards models were employed to examine the relationship between smoking, nutritional status and OS, with results presented as hazard ratios (HRs) and 95% confidence intervals (CIs). Multivariable logistic regression analyses were used to explore the relationship between smoking and malnutrition in cancer patients, with results presented as odds ratios (ORs) and 95% CIs. Univariable and multivariable Cox proportional hazards models were used to analyse haematological markers influencing OS in cancer patients. The box plot was used to illustrate the distribution differences in inflammatory markers' concentrations across different PG‐SGA scores and smoking durations. To assess the reliability of the results, sensitivity analyses were conducted. All statistical analyses were conducted using R software, Version 4.2.0 (R Project for Statistical Computing). A two‐sided *p*‐value of less than 0.05 was regarded as statistically significant.

## 3 Results

### 3.1 Basic Characteristics of Patients With Solid Tumours

A total of 29 988 patients with solid tumours were included in this study (Figure S1). Among them, 8200 were non‐smokers with no malnutrition, 9398 were non‐smokers with malnutrition, 4741 were smokers with no malnutrition, and 7649 were smokers with malnutrition (Table 1). In the smoking group, males accounted for more than 90% (91.8% in the no malnutrition group, 92.8% in the malnutrition group), whereas in the non‐smoking group, females made up more than 50% (74.5% in the no malnutrition group, 68.7% in the malnutrition group). In the smoking group, the average age of patients with malnutrition was significantly higher than that of those without malnutrition (60.17 years vs. 56.95 years), and the overall age of the smoking group was higher than that of the non‐smoking group. Regardless of smoking status, the average age of patients with malnutrition was higher than that of those without malnutrition.

**TABLE 1:** Baseline characteristics of patients with solid tumours, stratified by smoking status and nutritional condition.

Characteristics	Patients, no. (%) No smoking (*N* = 17 598)		Patients, no. (%) Smoking (*N* = 12 390)		*p*
	No malnutrition (*N* = 8200)	Malnutrition (*N* = 9398)	No malnutrition (*N* = 4741)	Malnutrition (*N* = 7649)	
Sex					
Male	2093 (25.5)	2944 (31.3)	4350 (91.8)	7095 (92.8)	< 0.001
Female	6107 (74.5)	6454 (68.7)	391 (8.2)	554 (7.2)	
Age (mean [SD])	53.10 (11.45)	57.13 (12.41)	56.95 (10.31)	60.17 (10.57)	< 0.001
Smoking					
No	8200 (100.0)	9398 (100.0)	0 (0.0)	0 (0.0)	< 0.001
Yes	0 (0.0)	0 (0.0)	4741 (100.0)	7649 (100.0)	
Packs (median [IQR])	0.00 [0.00, 0.00]	0.00 [0.00, 0.00]	1.00 [0.57, 1.00]	1.00 [0.57, 1.00]	< 0.001
Duration in years (median [IQR])	0.00 [0.00, 0.00]	0.00 [0.00, 0.00]	30.00 [20.00, 40.00]	30.00 [20.00, 40.00]	< 0.001
Pack‐year (median [IQR])	0.00 [0.00, 0.00]	0.00 [0.00, 0.00]	30.00 [14.29, 40.00]	30.00 [15.00, 42.86]	< 0.001
Cancer types					
Nasopharyngeal carcinoma	747 (9.1)	371 (3.9)	621 (13.1)	413 (5.4)	< 0.001
Digestive system cancers	2195 (26.8)	4880 (51.9)	1617 (34.1)	4372 (57.2)	
Lung cancer	1487 (18.1)	1713 (18.2)	1991 (42.0)	2522 (33.0)	
Breast cancer	2380 (29.0)	1069 (11.4)	116 (2.4)	91 (1.2)	
Genitourinary system cancers	1319 (16.1)	1281 (13.6)	361 (7.6)	208 (2.7)	
Malignant brain tumours	72 (0.9)	84 (0.9)	35 (0.7)	43 (0.6)	
TNM^a^					
I	1139 (16.8)	974 (12.7)	485 (12.6)	584 (9.4)	< 0.001
II	2001 (29.6)	1771 (23.1)	800 (20.7)	1207 (19.5)	
III	2011 (29.7)	2441 (31.8)	1336 (34.6)	2107 (34.0)	
IV	1613 (23.8)	2489 (32.4)	1241 (32.1)	2293 (37.0)	
Surgery					
No	6250 (76.2)	6821 (72.6)	3704 (78.1)	5544 (72.5)	< 0.001
Yes	1950 (23.8)	2577 (27.4)	1037 (21.9)	2105 (27.5)	
Chemotherapy					
No	3833 (46.7)	4731 (50.3)	2044 (43.1)	3871 (50.6)	< 0.001
Yes	4367 (53.3)	4667 (49.7)	2697 (56.9)	3778 (49.4)	
Radiotherapy					
No	7214 (88.0)	8448 (89.9)	4074 (85.9)	6725 (87.9)	< 0.001
Yes	986 (12.0)	950 (10.1)	667 (14.1)	924 (12.1)	
BMI (mean [SD])	23.62 (3.26)	21.87 (3.48)	23.31 (3.15)	21.47 (3.27)	< 0.001
PG‐SGA					
< 4	8200 (100.0)	0 (0.0)	4741 (100.0)	0 (0.0)	< 0.001
≥ 4	0 (0.0)	9398 (100.0)	0 (0.0)	7649 (100.0)	
COPD					
No	8180 (99.8)	9350 (99.5)	4663 (98.4)	7513 (98.2)	< 0.001
Yes	20 (0.2)	48 (0.5)	78 (1.6)	136 (1.8)	
Diabetes					
No	7681 (93.7)	8624 (91.8)	4338 (91.5)	7051 (92.2)	< 0.001
Yes	519 (6.3)	774 (8.2)	403 (8.5)	598 (7.8)	
Hypertension					
No	7036 (85.8)	7637 (81.3)	3921 (82.7)	6260 (81.8)	< 0.001
Yes	1164 (14.2)	1761 (18.7)	820 (17.3)	1389 (18.2)	
Occupation					
Mental labour	1221 (14.9)	1088 (11.6)	624 (13.2)	858 (11.2)	< 0.001
Physical labour	2489 (30.4)	3373 (35.9)	1742 (36.7)	2947 (38.5)	
Retirement and other	4490 (54.8)	4937 (52.5)	2375 (50.1)	3844 (50.3)	

*Note:* Digestive system cancers included oesophagus cancer, gastric cancer, colorectal cancer, liver cancer, carcinoma of biliary tract and pancreatic cancer. Genitourinary system cancers included cervical cancer, endometrial cancer, ovarian cancer, prostate cancer and carcinoma of bladder.

^a^ TNM: 5496 data missing.

### 3.2 Smoking and Malnutrition

Figure S2 showed that OS decreased as PG‐SGA scores increased in cancer patients. Figure S3 showed that longer smoking duration was associated with worse OS in cancer patients, and the restricted cubic spline regression curve illustrated a clear dose–response relationship. In Model 2 of Table 2, compared to the reference group of non‐smokers with no malnutrition (HR = 1), the death risk for non‐smokers with malnutrition increased by 25% (HR = 1.25, 95% CI 1.17–1.33, *p* < 0.001); for smokers with no malnutrition, the death risk increased by 24% (HR = 1.24, 95% CI 1.15–1.35, *p* < 0.001); and for smokers with malnutrition, the death risk increased by 46% (HR = 1.46, 95% CI 1.36–1.57, *p* < 0.001). Similar results were observed in both males and females. Table S1 revealed that compared to non‐smokers, smokers had a higher incidence of malnutrition (OR = 1.15, 95% CI 1.08–1.24, *p* < 0.001), and this result was observed in both males and females. Among the smokers, the threshold for smoking packs was 1, and for smoking duration, it was 22 (Figure S5). Due to fewer female smokers, a separate analysis was done for the female group, where the threshold for smoking packs was 0.1 and the threshold for smoking duration was 39 (Figure S6). The risk of malnutrition for short smoking duration and heavy packs increased significantly by 13% compared to the reference group (non‐smokers) (OR = 1.13, 95% CI 1.01–1.27, *p* = 0.033), whereas the risk for long duration and light packs showed a higher increase (OR = 1.19, 95% CI 1.06–1.34, *p* = 0.003), suggesting that long‐term smoking has a more lasting impact on malnutrition (Table S2). Long‐term smoking posed a greater risk for malnutrition in females (OR = 3.68, 95% CI 1.26–13.46, *p* = 0.027).

**TABLE 2:** The association between smoking status, nutritional condition and outcomes stratified by sex.

All	Model 0			Model 1			Model 2	
	HR (95% CI)	*p*		HR (95% CI)	*p*		HR (95% CI)	*p*
No smoking and no malnutrition	1 [reference]		No smoking and no malnutrition			No smoking and no malnutrition		
No smoking and malnutrition	1.39 (1.32, 1.46)	< 0.001	No smoking and malnutrition	1.26 (1.19, 1.34)	< 0.001	No smoking and malnutrition	1.25 (1.17, 1.33)	< 0.001
Smoking and no malnutrition	1.55 (1.45, 1.65)	< 0.001	Smoking and no malnutrition	1.25 (1.16, 1.35)	< 0.001	Smoking and no malnutrition	1.24 (1.15, 1.35)	< 0.001
Smoking and malnutrition	1.93 (1.83, 2.04)	<0.001	Smoking and malnutrition	1.49 (1.38, 1.59)	< 0.001	Smoking and malnutrition	1.46 (1.36, 1.57)	< 0.001
Male								
No smoking and no malnutrition	1 [Reference]		No smoking and no malnutrition			No smoking and no malnutrition		
No smoking and malnutrition	1.32 (1.2, 1.45)	< 0.001	No smoking and malnutrition	1.28 (1.16, 1.43)	< 0.001	No smoking and malnutrition	1.27 (1.14, 1.41)	< 0.001
Smoking and no malnutrition	1.27 (1.16, 1.38)	< 0.001	Smoking and no malnutrition	1.22 (1.11, 1.35)	< 0.001	Smoking and no malnutrition	1.22 (1.1, 1.35)	< 0.001
Smoking and malnutrition	1.57 (1.45, 1.7)	< 0.001	Smoking and malnutrition	1.46 (1.33, 1.6)	< 0.001	Smoking and malnutrition	1.44 (1.31, 1.58)	< 0.001
Female								
No smoking and no malnutrition	1 [Reference]		No smoking and no malnutrition			No smoking and no malnutrition		
No smoking and malnutrition	1.4 (1.31, 1.49)	< 0.001	No smoking and malnutrition	1.23 (1.15, 1.33)	< 0.001	No smoking and malnutrition	1.22 (1.13, 1.32)	< 0.001
Smoking and no malnutrition	1.43 (1.18, 1.74)	< 0.001	Smoking and no malnutrition	1.33 (1.06, 1.67)	0.012	Smoking and no malnutrition	1.32 (1.06, 1.66)	0.015
Smoking and malnutrition	2.01 (1.74, 2.32)	< 0.001	Smoking and malnutrition	1.71 (1.45, 2.01)	< 0.001	Smoking and malnutrition	1.71 (1.45, 2.01)	< 0.001

*Note:* Model 0 was not adjusted for any covariates. Model 1 was adjusted for sex, age, TNM and cancer types. Model 2 was adjusted for sex, age, TNM, cancer types, surgery, chemotherapy, radiotherapy, occupation and BMI.

### 3.3 The Association Between Smoking Duration and OS

Figure S7 showed that, among smoking cancer patients, OS gradually decreased with increasing smoking duration. Table S3 was the baseline table for patients based on the smoking duration tertiles (T1: 0–20, T2: 20–32, T3: ≥ 32). Among smokers with solid tumours, each 5‐year increase in smoking duration was associated with a 6% higher risk of death (HR = 1.06, 95% CI 1.04, 1.07, *p* < 0.001); the risk for the high‐duration group (≥ 22 years) was 31% higher compared to the reference group (HR = 1.31, 95% CI 1.21–1.41, *p* < 0.001); using T1 group as the reference, the risk for T3 group increased by 40% (HR = 1.40, 95% CI 1.26–1.57, *p* < 0.001), and trend analysis confirmed that the risk increased with longer smoking duration (*p* for trend < 0.001). These results held true for both males and females (Table S4). Figure S8 showed that longer smoking duration was associated with progressively shorter OS among participants with malnutrition. Figure S9 showed that in participants with malnutrition, the threshold value for smoking duration was 25. Table S5 was the baseline table for patients based on smoking duration tertiles (T1: 0, T2: 0–20, T3: ≥ 20). Table S6 presented the analysis of the relationship between smoking duration and OS in malnourished patients with solid tumours. The findings indicated that regardless of covariate adjustments, longer smoking duration significantly increased the death risk in these patients. Specifically, in the fully adjusted model (Model 2), each additional 10 years of smoking was associated with an 86% higher risk of death (HR = 1.86, 95% CI 1.55–2.23, *p* < 0.001). When grouped by smoking duration, the long‐duration group (≥ 25 years) had a significantly higher death risk compared to the reference group (HR = 1.24, 95% CI 1.16–1.32, *p* < 0.001), and when T1 group was used as a reference, the risk in the T3 group increased by 25% (HR = 1.25, 95% CI 1.16–1.35, *p* < 0.001). Gender stratification showed that this increased risk was present in both male and female patients, with a more significant risk increase in females. Sensitivity analysis also confirmed the robustness of the above results (Tables S11 and S12).

### 3.4 The Connection Between Smoking, Malnutrition and Inflammation

**FIGURE 1:**
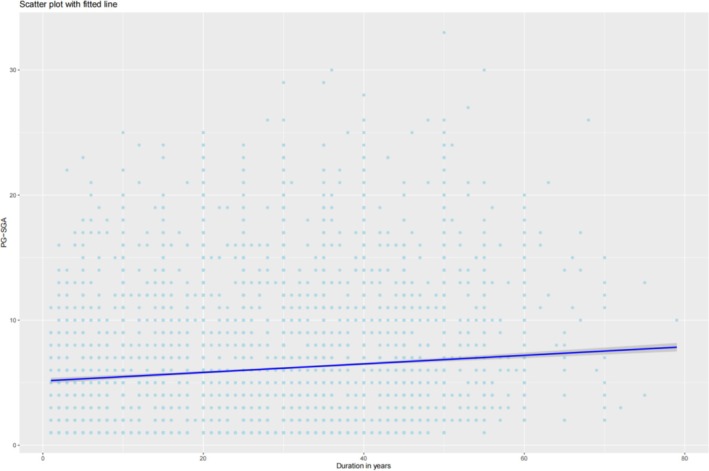
Scatter plot with fitted line of PG‐SGA and duration in years in patients with solid tumours.

**FIGURE 2:**
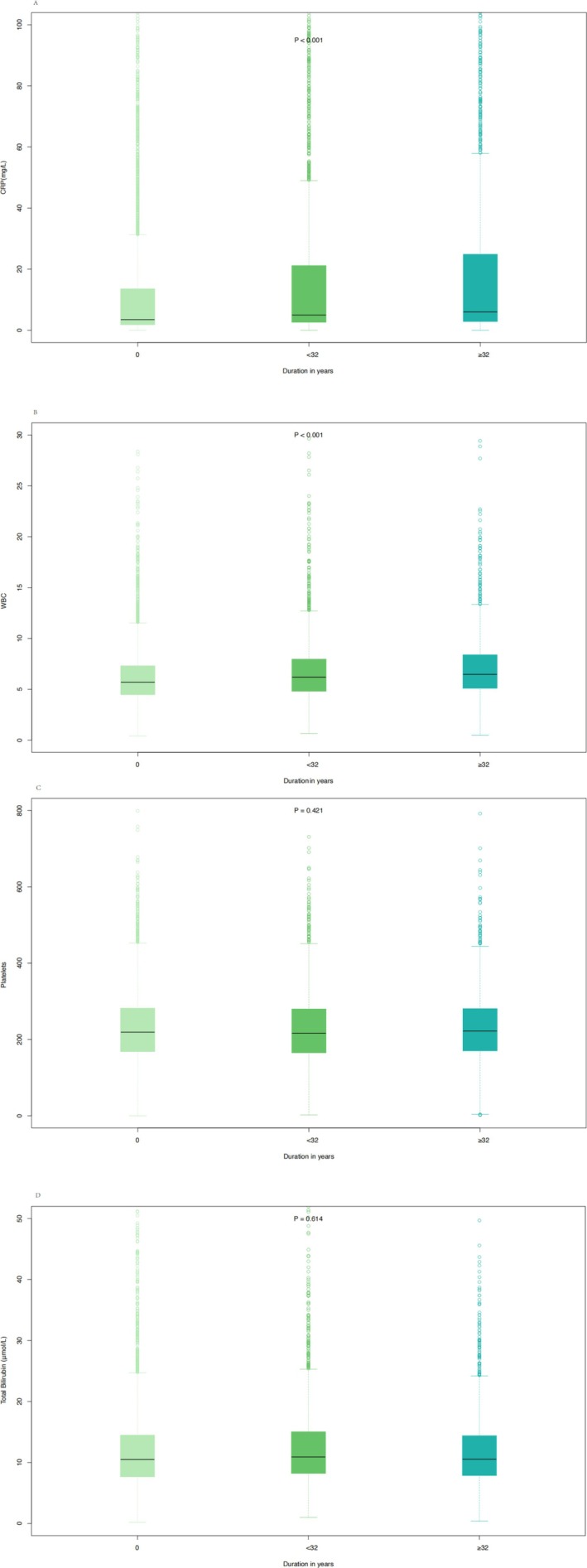
The box plot of haematological indicators in smoking patients.

**FIGURE 3:**
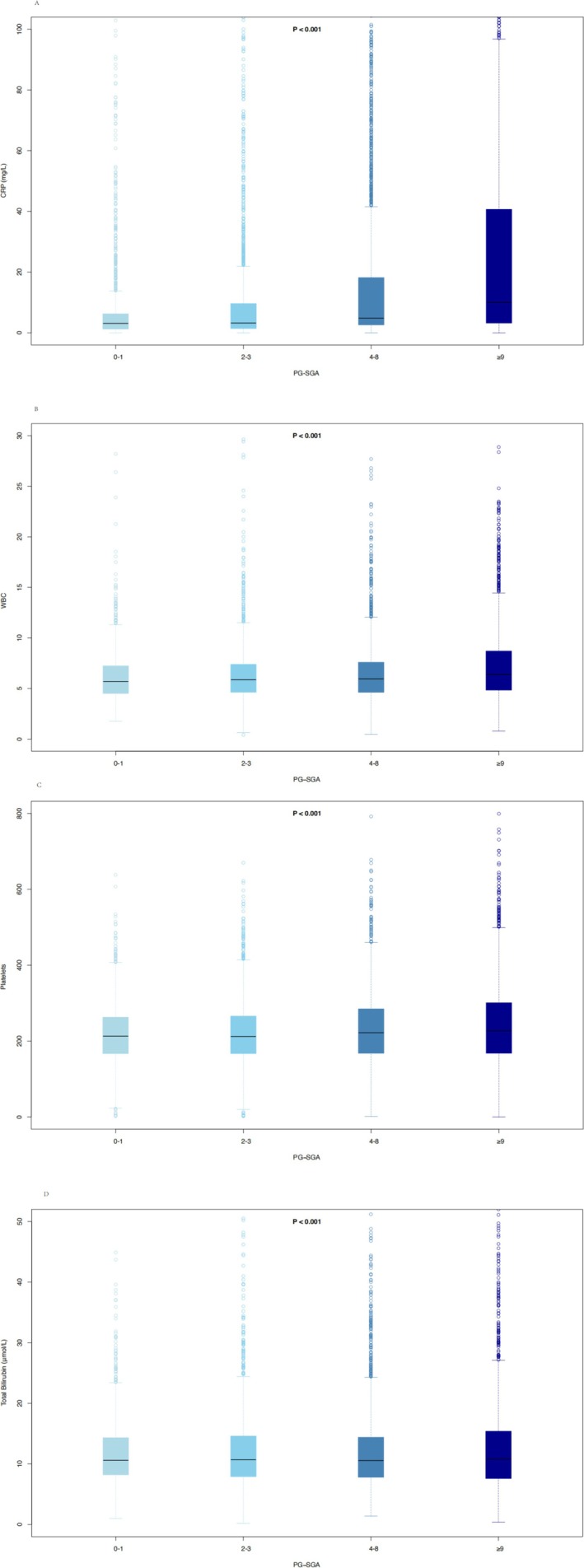
The box plot of haematological indicators in malnutrition patients.

Figure 1 illustrated that longer smoking duration was associated with higher PG‐SGA scores. Figure S10 indicated that 9065 patients with complete haematological data were included. Table S7 presented baseline characteristics stratified by smoking duration tertiles, revealing a significant increase in median CRP levels (from 3.50 to 6.00 mg/L) and a rising trend in WBC count (from 5.70 × 10^9^/L to 6.47 × 10^9^/L) with increasing smoking duration. Table S8 summarized the baseline characteristics according to PG‐SGA categories and showed that as nutritional status worsened, both CRP and WBC levels increased markedly (CRP from 3.11 to 10.00 mg/L; WBC from 5.70 × 10^9^/L to 6.39 × 10^9^/L). In smoking populations, univariate and multivariate Cox regression analyses (Table S9) identified CRP, WBC, platelet count and total bilirubin as risk factors for OS. Similarly, in malnourished populations, Cox regression analyses (Table S10) demonstrated that albumin, CRP, WBC, platelet count and total bilirubin were risk factors for OS. Figure 2 showed that with increasing smoking duration, CRP levels (median [IQR]) rose progressively (3.50 [1.79–13.60]; 5.00 [2.60–21.20]; 6.00 [2.87–24.90]), and WBC levels increased correspondingly (5.70 [4.47–7.31]; 6.20 [4.80–7.97]; 6.47 [5.10–8.40]), whereas platelet count and total bilirubin showed no significant increasing trend across smoking groups. Figure 3 demonstrated that higher PG‐SGA scores were associated with significantly elevated CRP (3.11 [1.23–6.28]; 3.23 [1.40–9.62]; 4.83 [2.60–18.20]; 10.00 [3.20–40.70]) and WBC levels (5.70 [4.51–7.24]; 5.88 [4.63–7.40]; 5.95 [4.62–7.60]; 6.39 [4.84–8.71]), whereas platelet count and bilirubin concentrations showed no significant increasing trend. Mediation analyses (Figure S12) indicated that CRP accounted for 4.7% and WBC for 6.3% of the association between smoking and mortality. In the association between malnutrition and mortality (Figure S13), CRP mediated 9.1% and WBC mediated 4.4%.

## 4 Discussion

This study comprehensively examined the joint effects of smoking and malnutrition on OS in patients with solid tumours, addressing limitations of previous single‐factor analyses. Using a large cohort of nearly 30 000 patients, we demonstrated that both smoking and malnutrition independently predicted worse prognosis, and the highest risk of mortality was observed when both were present. A clear dose–response relationship was observed for smoking duration, and inflammatory markers such as CRP and WBC partially mediated these associations. These findings highlight the synergistic role of smoking and malnutrition in promoting systemic inflammation and adverse clinical outcomes.

The results indicated that both smoking and malnutrition independently raised the death risk, and their combination resulted in an even higher death risk. These findings are consistent with prior research. Phua et al. found that, compared to those who never smoked, both former and current smokers had an elevated risk of smoking‐related second primary cancers [32]. Park et al. found that by effectively controlling smoking at both the population and individual levels in Korean men, around one‐third of cancer deaths could be prevented [33]. Smoking, recognized as one of the primary risk factors for cancer, has been extensively proven to elevate the risk of cancer and decrease the treatment effectiveness and survival rates of cancer patients [33, 34].

### 4.1 Regarding the Association Between Smoking and Overall Mortality

We found that longer smoking duration was associated with an increased risk of death, showing a clear dose–response relationship. Patients with extended smoking histories exhibited significantly higher mortality, suggesting that long‐term smoking served as an adverse prognostic factor in cancer survival. This finding is consistent with previous studies. Kwan et al. found that longer duration and more pack‐years of cigarette smoking were associated with a higher risk of recurrence of non‐muscle‐invasive bladder cancer [35]. Doll et al. found that among men born around 1920, long‐term smoking in adulthood doubled the mortality rate in specific age groups, but quitting smoking at age 50 reduced this risk by half, and quitting at age 30 could nearly eliminate the risk [36].

The role of inflammation in cancer progression has been widely studied. Our research found that both smoking duration and malnutrition were associated with elevated inflammation markers such as CRP and WBC count, suggesting that inflammation may be one of the mechanisms by which smoking and poor nutritional status jointly shorten survival. Smoking‐related oxidative stress can exacerbate the inflammatory response, further promoting cancer development and progression [21]. Myburgh et al. found that active smokers with the rs3093068 C allele had an increased risk of elevated CRP concentrations [37]. Berania et al. found that active smokers had significantly higher WBC counts compared to never smokers and former smokers [38]. The development of cancer malnutrition also causes a further increase in inflammation levels in the body. Gomes de Lima et al. found that patients with weight loss and malnutrition had significantly higher serum CRP levels compared to patients without weight loss and in good nutritional status [39]. Xie et al. found that a high IBI (C‐reactive protein × neutrophil/lymphocyte ratio) is an independent high‐risk factor influencing cancer patients' physical condition, malnutrition, cachexia and short‐term prognosis [40]. Therefore, shortening smoking duration and improving nutritional status may help reduce tumour‐related inflammation levels and enhance patient survival.

The impact of smoking and malnutrition on the OS of cancer patients has been reported in many studies. However, this study provides a detailed and in‐depth investigation of the combined effect of smoking (packs, duration in years, pack‐year) and malnutrition on the OS of cancer patients and explores the underlying mechanism of increased inflammation levels, guiding clinical control of inflammation in cancer patients with smoking and malnutrition. These findings suggest that cancer patients with both smoking exposure and poor nutritional status may particularly benefit from anti‐inflammatory interventions, highlighting the need for targeted management strategies in this high‐risk population. The limitations are as follows: (1) Haematological data were limited, and other inflammatory markers related to smoking or malnutrition still needed to be investigated. (2) The biological mechanisms of inflammation underlying smoking and malnutrition required further exploration.

## 5 Conclusion

Smoking and malnutrition are important factors affecting the survival of cancer patients. Our findings highlight the importance of intervening in smoking duration and improving nutritional status in the management of cancer patients. It also guides clinical control of CRP and WBC inflammation levels in smokers with malnutrition.

## Funding

This work was supported by the National Key Research and Development Program (2022YFC2009600, 2022YFC2009601), Laboratory for Clinical Medicine, Capital Medical University (2023‐SYJCLC01) and Nation Multidisciplinary Cooperative Diagnosis and Treatment Capacity Project for Major Diseases: Comprehensive Treatment and Management of Critically Ill Elderly Inpatients (No. 2019.YLFW) to Dr Hanping Shi.

## Ethics Statement

The study was conducted in accordance with the principles of the Declaration of Helsinki. All participating centers and institutions received approval from the relevant Ethics Committee (Registration No.: ChiCTR1800020329; Ethics approval number: Medical Research Ethics Review [2013] 82). The Ethics Committee overseeing the study was the Medical Ethics Committee of the First Affiliated Hospital of Sun Yat‐sen University.

## Conflicts of Interest

The authors declare no conflicts of interest.

## Disclaimer

The views and conclusions expressed in this study are solely those of the authors and do not necessarily reflect the official policies or positions of the affiliated institutions or funding agencies. This research was conducted independently, and any opinions, findings or conclusions presented are the authors' own.

## Supporting information


**Figure S1:** Flow chart.
**Figure S2:** Association between PG‐SGA scores and all‐cause mortality in patients with solid tumours using a restricted cubic spline regression model among all participants.
**Figure S3:** Association between smoking and all‐cause mortality in patients with solid tumours using a restricted cubic spline regression model among all participants.
**Figure S4:** Prevalence rate of cancer types.
**Figure S5:** Cut‐off value calculation among all smoking participants.
**Figure S6:** Cut‐off value calculation among all smoking female participants.
**Table S1:** The association between smoking status and nutritional condition stratified by sex.
**Table S2:** The association between smoking status and nutritional condition stratified by sex.
**Figure S7:** Association between smoking and all‐cause mortality in patients with solid tumours using a restricted cubic spline regression model among all smoking participants.
**Table S3:** Baseline characteristics of patients with solid tumours among smoking participants, stratified by smoking duration tertiles.
**Table S4:** The association of duration in years with all‐cause mortality in smoking patients with solid tumours.
**Figure S8:** Association between smoking and all‐cause mortality in patients with solid tumours using a restricted cubic spline regression model among all malnutrition participants.
**Figure S9:** Cut‐off value calculation among all malnutrition participants.
**Table S5:** Baseline characteristics of patients with solid tumours among malnutrition participants, stratified by smoking duration tertiles.
**Table S6:** The association of duration in years with all‐cause mortality in malnutrition patients with solid tumours.
**Figure S10:** Flow chart.
**Figure S11:** Cut‐off value calculation among all smoking participants.
**Table S7:** Baseline characteristics of patients with solid tumours, stratified by smoking duration cut‐off values.
**Table S8:** Baseline characteristics of patients with solid tumours, stratified by PG‐SGA score values.
**Table S9:** Univariate and multivariate Cox regression analyses of haematological indicators in smoking populations.
**Table S10:** Univariate and multivariate Cox regression analyses of haematological indicators in malnutrition populations.
**Table S11:** Sensitivity analysis of the association between smoking duration and all‐cause mortality in Smoking Patients.
**Table S12:** Sensitivity analysis of the association between smoking duration and all‐cause mortality in malnutrition patients.
**Figure S12:** Hazard ratios (HR) for all‐cause mortality with 95% confidence intervals are shown for smoking in relation to CRP and WBC.
**Figure S13:** Hazard ratios (HR) for all‐cause mortality with 95% confidence intervals are shown for PG‐SGA in relation to CRP and WBC.

## Data Availability

The datasets used and/or analysed in the current study are available from the corresponding author upon reasonable request.

## References

[1] S. Kiri and T. Ryba , “Cancer, Metastasis, and the Epigenome,” Molecular Cancer 23, no. 1 (2024): 154. 39095874 10.1186/s12943-024-02069-w PMC11295362

[2] R. L. Siegel , A. N. Giaquinto , and A. Jemal , “Cancer Statistics, 2024,” CA: A Cancer Journal for Clinicians 74, no. 1 (2024): 12–49. 38230766 10.3322/caac.21820

[3] D. Sun , H. Li , M. Cao , et al., “Cancer Burden in China: Trends, Risk Factors and Prevention,” Cancer Biology & Medicine 17, no. 4 (2020): 879–895. 33299641 10.20892/j.issn.2095-3941.2020.0387 PMC7721090

[4] S. M. Schwartz , “Epidemiology of Cancer,” Clinical Chemistry 70, no. 1 (2024): 140–149. 38175589 10.1093/clinchem/hvad202

[5] N. Adler , A. T. Bahcheli , K. C. L. Cheng , et al., “Mutational Processes of Tobacco Smoking and APOBEC Activity Generate Protein‐Truncating Mutations in Cancer Genomes,” Science Advances 9, no. 44 (2023): eadh3083. 37922356 10.1126/sciadv.adh3083 PMC10624356

[6] H. Scherübl , “Smoking Tobacco and Cancer Risk,” Deutsche Medizinische Wochenschrift 146, no. 6 (2021): 412–417. 33735927 10.1055/a-1216-7050

[7] N. L. Benowitz , “Nicotine Addiction,” New England Journal of Medicine 362, no. 24 (2010): 2295–2303. 20554984 10.1056/NEJMra0809890 PMC2928221

[8] S. Chellappan , “Smoking Cessation After Cancer Diagnosis and Enhanced Therapy Response: Mechanisms and Significance,” Current Oncology 29, no. 12 (2022): 9956–9969. 36547196 10.3390/curroncol29120782 PMC9776692

[9] R. E. Gemine , G. R. Davies , K. Lanyon , S. E. Rees , I. Campbell , and K. E. Lewis , “Quitting Smoking Improves Two‐Year Survival After a Diagnosis of Non‐Small Cell Lung Cancer,” Lung Cancer 186 (2023): 107388. 37820539 10.1016/j.lungcan.2023.107388

[10] J. T. Chang , G. M. Anic , B. L. Rostron , M. Tanwar , and C. M. Chang , “Cigarette Smoking Reduction and Health Risks: A Systematic Review and Meta‐Analysis,” Nicotine & Tobacco Research 23, no. 4 (2021): 635–642. 32803250 10.1093/ntr/ntaa156

[11] M. Morton , J. Patterson , J. Sciuva , et al., “Malnutrition, Sarcopenia, and Cancer Cachexia in Gynecologic Cancer,” Gynecologic Oncology 175 (2023): 142–155. 37385068 10.1016/j.ygyno.2023.06.015

[12] A. F. Bullock , S. L. Greenley , G. A. G. McKenzie , L. W. Paton , and M. J. Johnson , “Relationship Between Markers of Malnutrition and Clinical Outcomes in Older Adults With Cancer: Systematic Review, Narrative Synthesis and Meta‐Analysis,” European Journal of Clinical Nutrition 74, no. 11 (2020): 1519–1535. 32366995 10.1038/s41430-020-0629-0 PMC7606134

[13] J. Cao , H. Xu , W. Li , et al., “Nutritional Assessment and Risk Factors Associated to Malnutrition in Patients With Esophageal Cancer,” Current Problems in Cancer 45, no. 1 (2021): 100638. 32829957 10.1016/j.currproblcancer.2020.100638

[14] A. F. Da Ré , L. G. Gurgel , G. Buffon , W. E. R. Moura , D. C. G. Marques Vidor , and M. A. P. Maahs , “Tobacco Influence on Taste and Smell: Systematic Review of the Literature,” International Archives of Otorhinolaryngology 22, no. 1 (2018): 81–87. 29371903 10.1055/s-0036-1597921 PMC5783692

[15] S. Yuan and S. C. Larsson , “Epidemiology of Sarcopenia: Prevalence, Risk Factors, and Consequences,” Metabolism 144 (2023): 155533. 36907247 10.1016/j.metabol.2023.155533

[16] K. B. Kennel , M. Bozlar , A. F. De Valk , and F. R. Greten , “Cancer‐Associated Fibroblasts in Inflammation and Antitumor Immunity,” Clinical Cancer Research 29, no. 6 (2023): 1009–1016. 36399325 10.1158/1078-0432.CCR-22-1031 PMC10011884

[17] C. I. Diakos , K. A. Charles , D. C. McMillan , and S. J. Clarke , “Cancer‐Related Inflammation and Treatment Effectiveness,” Lancet Oncology 15, no. 11 (2014): e493–e503. 25281468 10.1016/S1470-2045(14)70263-3

[18] L. M. Coussens and Z. Werb , “Inflammation and Cancer,” Nature 420, no. 6917 (2002): 860–867. 12490959 10.1038/nature01322 PMC2803035

[19] A. Mantovani , P. Allavena , A. Sica , and F. Balkwill , “Cancer‐Related Inflammation,” Nature 454, no. 7203 (2008): 436–444. 18650914 10.1038/nature07205

[20] A. W. Caliri , S. Tommasi , and A. Besaratinia , “Relationships Among Smoking, Oxidative Stress, Inflammation, Macromolecular Damage, and Cancer,” Mutation Research, Reviews in Mutation Research 787 (2021): 108365. 34083039 10.1016/j.mrrev.2021.108365 PMC8287787

[21] Y. Liu , L. Lu , H. Yang , et al., “Dysregulation of Immunity by Cigarette Smoking Promotes Inflammation and Cancer: A Review,” Environmental Pollution 339 (2023): 122730. 37838314 10.1016/j.envpol.2023.122730

[22] A. Kaplan , E. Abidi , R. Ghali , G. W. Booz , F. Kobeissy , and F. A. Zouein , “Functional, Cellular, and Molecular Remodeling of the Heart Under Influence of Oxidative Cigarette Tobacco Smoke,” Oxidative Medicine and Cellular Longevity 2017 (2017): 3759186. 28808498 10.1155/2017/3759186 PMC5541812

[23] C. M. Best , K. Sun , S. de Pee , M. Sari , M. W. Bloem , and R. D. Semba , “Paternal Smoking and Increased Risk of Child Malnutrition Among Families in Rural Indonesia,” Tobacco Control 17, no. 1 (2008): 38–45. 18218806 10.1136/tc.2007.020875

[24] J. Chai , F. C. Chu , T. W. Chow , and N. C. Shum , “Prevalence of Malnutrition and Its Risk Factors in Stroke Patients Residing in an Infirmary,” Singapore Medical Journal 49, no. 4 (2008): 290–296. 18418520

[25] Ø. Bruserud , H. H. Aarstad , and T. H. A. Tvedt , “Combined C‐Reactive Protein and Novel Inflammatory Parameters as a Predictor in Cancer‐What Can We Learn From the Hematological Experience?,” Cancers (Basel) 12, no. 7 (2020): 1966. 32707721 10.3390/cancers12071966 PMC7409204

[26] J. M. Yu , M. Yang , H. X. Xu , et al., “Association Between Serum C‐Reactive Protein Concentration and Nutritional Status of Malignant Tumor Patients,” Nutrition and Cancer 71, no. 2 (2019): 240–245. 30450976 10.1080/01635581.2018.1524019

[27] C. O. Enwonwu and V. I. Meeks , “Bionutrition and Oral Cancer in Humans,” Critical Reviews in Oral Biology and Medicine 6, no. 1 (1995): 5–17. 7632867 10.1177/10454411950060010401

[28] H. Xu , C. Song , L. Yin , et al., “Extension Protocol for the Investigation on Nutrition Status and Clinical Outcome of Patients With Common Cancers in China (INSCOC) Study: 2021 Update,” Precision Nutrition 1, no. 2 (2022): e00014.

[29] M. E. Cornelius , C. G. Loretan , A. Jamal , et al., “Tobacco Product Use Among Adults – United States, 2021,” MMWR. Morbidity and Mortality Weekly Report 72, no. 18 (2023): 475–483. 37141154 10.15585/mmwr.mm7218a1 PMC10168602

[30] H. Jager‐Wittenaar and F. D. Ottery , “Assessing Nutritional Status in Cancer: Role of the Patient‐Generated Subjective Global Assessment,” Current Opinion in Clinical Nutrition and Metabolic Care 20, no. 5 (2017): 322–329. 28562490 10.1097/MCO.0000000000000389

[31] C. Song , K. Wang , Z. Guo , et al., “Investigation of Nutritional Status in Chinese Patients With Common Cancer,” Scientia Sinica Vitae 50, no. 12 (2020): 1437–1452.

[32] Z. J. Phua , R. J. MacInnis , and H. Jayasekara , “Cigarette Smoking and Risk of Second Primary Cancer: A Systematic Review and Meta‐Analysis,” Cancer Epidemiology 78 (2022): 102160. 35430427 10.1016/j.canep.2022.102160

[33] S. Park , S. H. Jee , H. R. Shin , et al., “Attributable Fraction of Tobacco Smoking on Cancer Using Population‐Based Nationwide Cancer Incidence and Mortality Data in Korea,” BMC Cancer 14 (2014): 406. 24902960 10.1186/1471-2407-14-406 PMC4090397

[34] G. W. Warren and K. M. Cummings , “Tobacco and Lung Cancer: Risks, Trends, and Outcomes in Patients With cancer,” in American Society of Clinical Oncology Educational Book (2013): 359–364. 10.14694/EdBook_AM.2013.33.359 23714547

[35] M. L. Kwan , R. Haque , K. C. Young‐Wolff , et al., “Smoking Behaviors and Prognosis in Patients With Non‐Muscle‐Invasive Bladder Cancer in the Be‐Well Study,” JAMA Network Open 5, no. 11 (2022): e2244430. 36449286 10.1001/jamanetworkopen.2022.44430 PMC9713602

[36] R. Doll , R. Peto , J. Boreham , and I. Sutherland , “Mortality in Relation to Smoking: 50 Years' Observations on Male British Doctors,” BMJ 328, no. 7455 (2004): 1519. 15213107 10.1136/bmj.38142.554479.AE PMC437139

[37] P. H. Myburgh , C. Nienaber‐Rousseau , I. M. Kruger , and G. W. Towers , “Education, Smoking and CRP Genetics in Relation to C‐Reactive Protein Concentrations in Black South Africans,” International Journal of Environmental Research and Public Health 17, no. 18 (2020): 6646. 32933066 10.3390/ijerph17186646 PMC7558133

[38] I. Berania , L. M. Endam , A. Filali‐Mouhim , et al., “Active Smoking Status in Chronic Rhinosinusitis Is Associated With Higher Serum Markers of Inflammation and Lower Serum Eosinophilia,” International Forum of Allergy & Rhinology 4, no. 5 (2014): 347–352. 24431239 10.1002/alr.21289

[39] K. V. Gomes de Lima and R. Maio , “Nutritional Status, Systemic Inflammation and Prognosis of Patients With Gastrointestinal Cancer,” Nutrición Hospitalaria 27, no. 3 (2012): 707–714. 23114934 10.3305/nh/2012.27.3.5567

[40] H. Xie , G. Ruan , Y. Ge , et al., “Inflammatory Burden as a Prognostic Biomarker for Cancer,” Clinical Nutrition 41, no. 6 (2022): 1236–1243. 35504166 10.1016/j.clnu.2022.04.019

