# Supplementary material for: Combined Impact of Smoking Duration and Malnutrition on Cancer Survival: Insights Into Systemic Inflammation
Source: J Cachexia Sarcopenia Muscle. 2026 Feb 17;17(1):e70207. doi: 10.1002/jcsm.70207 (PMC12913709; doi:10.1002/jcsm.70207)
Supplement: Supplementary file 1 — Figure S1: Flow chart. Figure S2: Association between PG‐SGA scores and all‐cause mortality in patients with solid tumours using a restricted cubic spline regression model among all participants. Figure S3: Association between smoking and all‐cause mortality in patients with solid tumours using a restricted cubic spline regression model among all participants. Figure S4: Prevalence rate of cancer types. Figure S5: Cut‐off value calculation among all smoking participants. Figure S6: Cut‐off value calculation among all smoking female participants. Table S1: The association between smoking status and nutritional condition stratified by sex. Table S2: The association between smoking status and nutritional condition stratified by sex. Figure S7: Association between smoking and all‐cause mortality in patients with solid tumours using a restricted cubic spline regression model among all smoking participants. Table S3: Baseline characteristics of patients with solid tumours among smoking participants, stratified by smoking duration tertiles. Table S4: The association of duration in years with all‐cause mortality in smoking patients with solid tumours. Figure S8: Association between smoking and all‐cause mortality in patients with solid tumours using a restricted cubic spline regression model among all malnutrition participants. Figure S9: Cut‐off value calculation among all malnutrition participants. Table S5: Baseline characteristics of patients with solid tumours among malnutrition participants, stratified by smoking duration tertiles. Table S6: The association of duration in years with all‐cause mortality in malnutrition patients with solid tumours. Figure S10: Flow chart. Figure S11: Cut‐off value calculation among all smoking participants. Table S7: Baseline characteristics of patients with solid tumours, stratified by smoking duration cut‐off values. Table S8: Baseline characteristics of patients with solid tumours, stratified by PG‐SGA score values. Table S9: Univari [file JCSM-17-e70207-s001.pdf]

Figure S1 : Flow Chart.

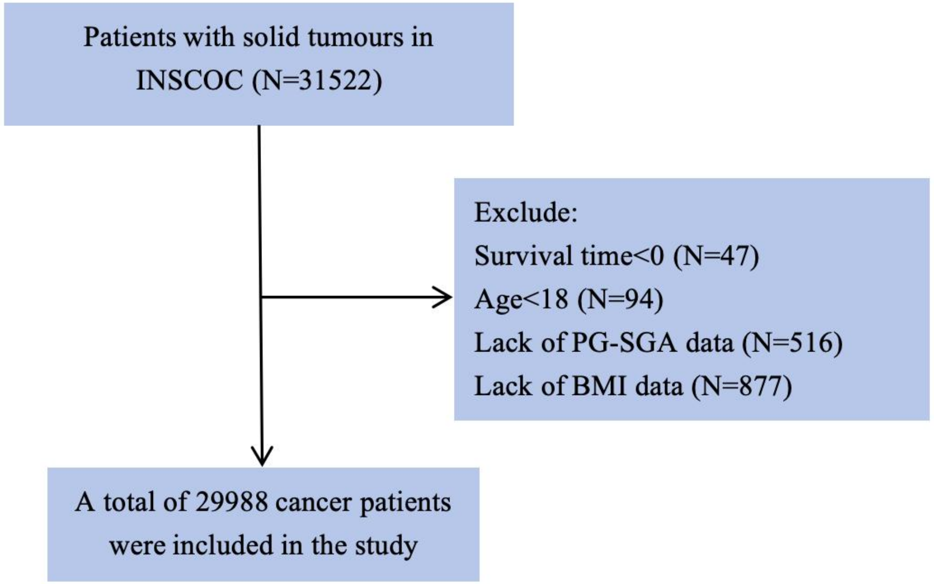

Figure S2 : Association between PG-SGA scores and all-cause mortality in patients with solid tumours using a restricted cubic spline regression model among all participants.

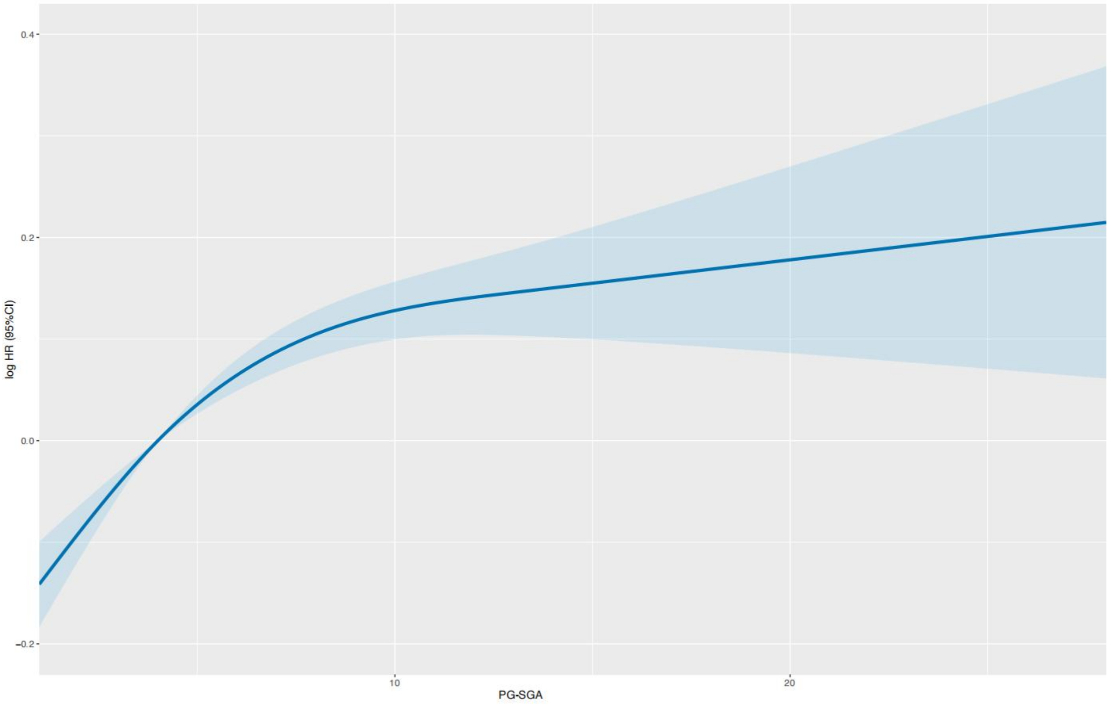

Model was adjusted for sex, age, TNM, cancer types, surgery, chemotherapy, radiotherapy, occupation, BMI.

Figure S3 : Association between smoking and all-cause mortality in patients with solid tumours using a restricted cubic spline regression model among all participants.

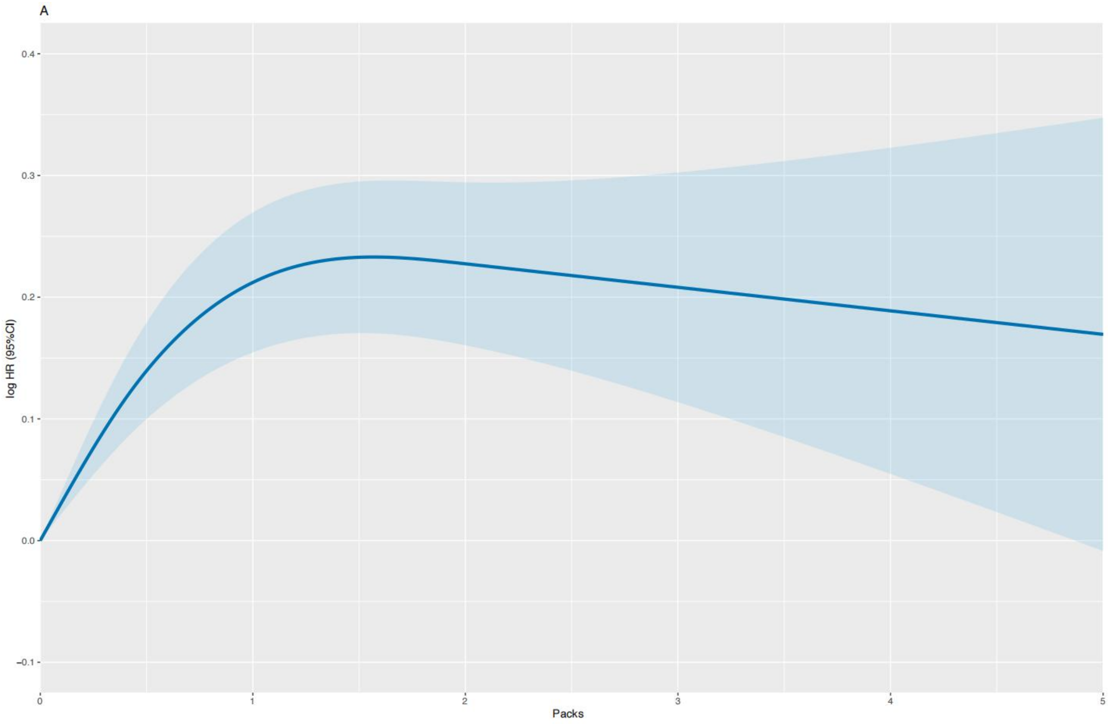

B

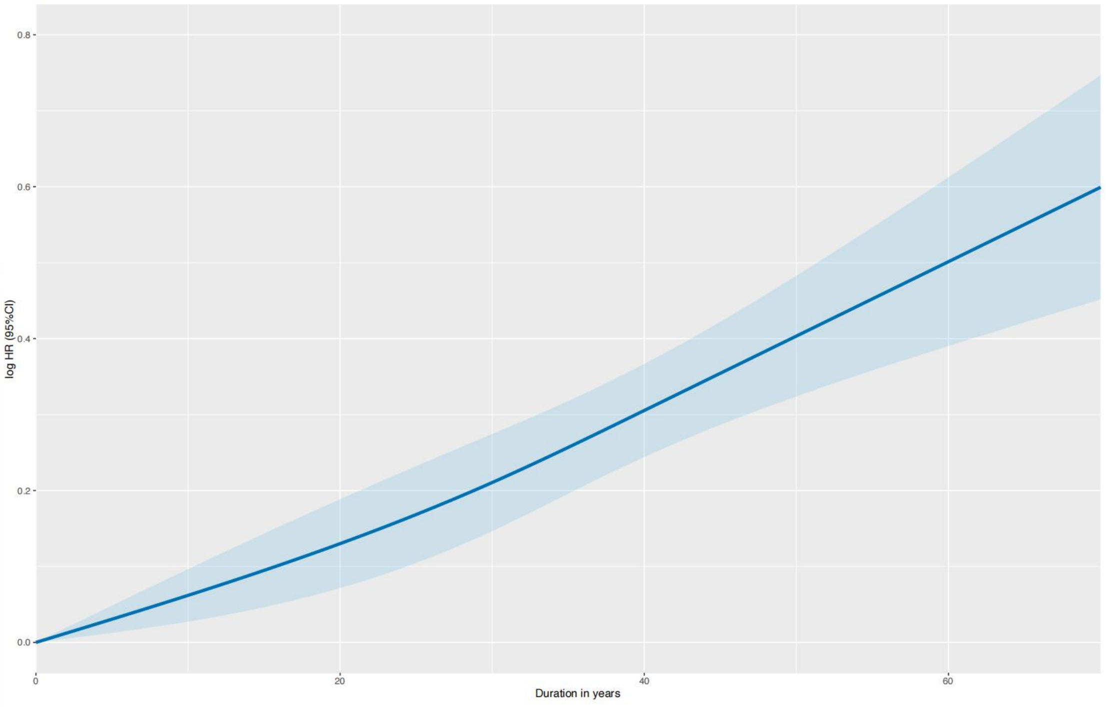

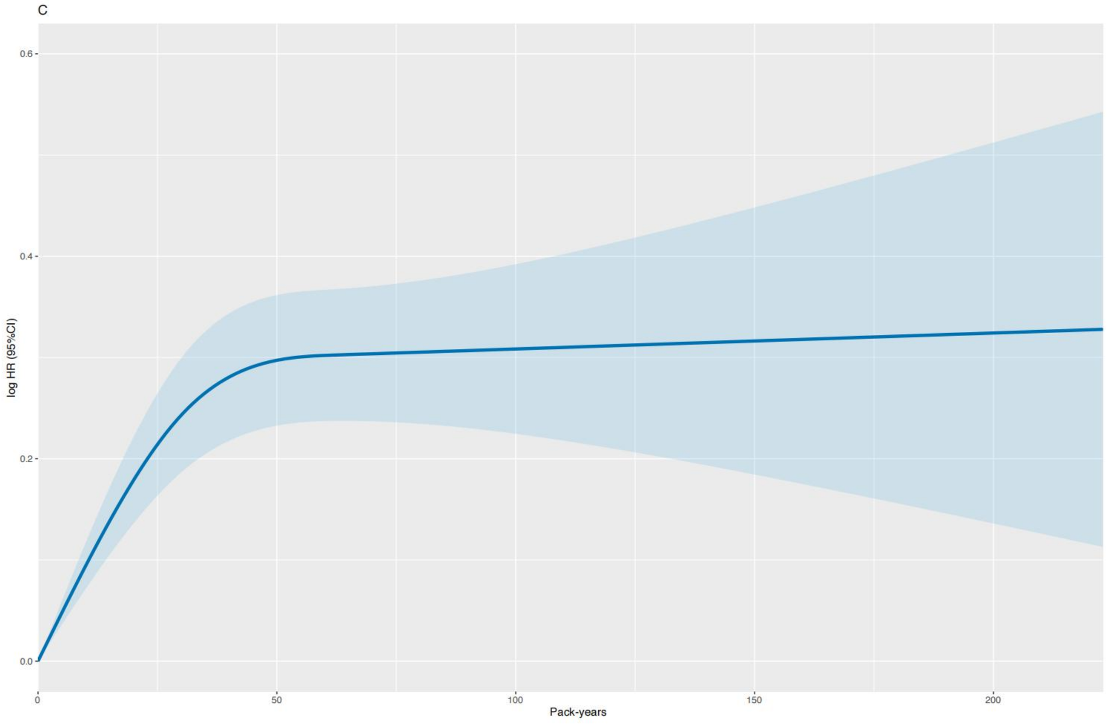

Model was adjusted for sex, age, TNM, cancer types, surgery, chemotherapy, radiotherapy, occupation, BMI.

Figure S4 : Prevalence Rate of Cancer Types.

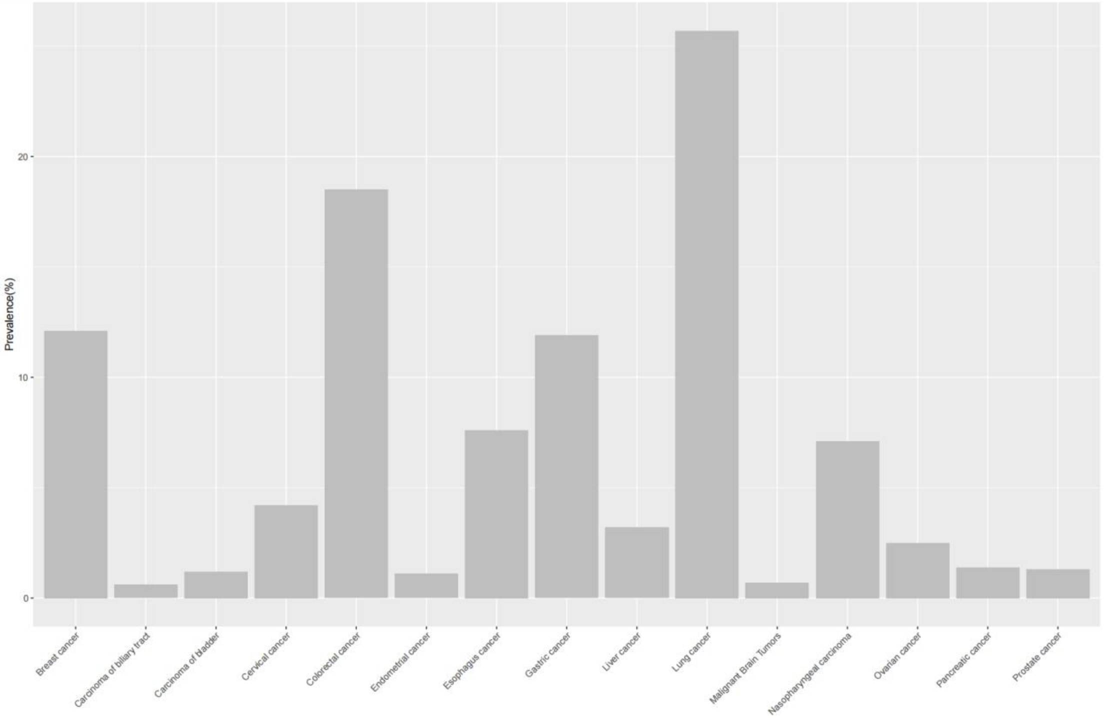

Figure S5 : Cutoff value calculation among all smoking participants.

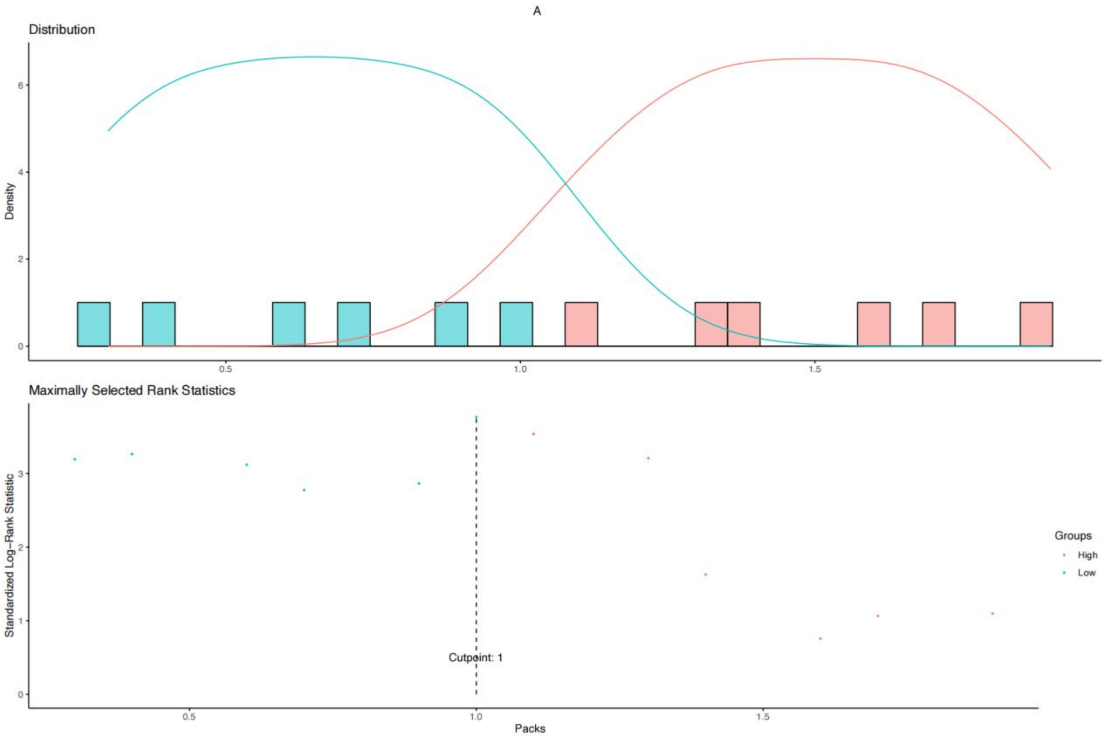

B

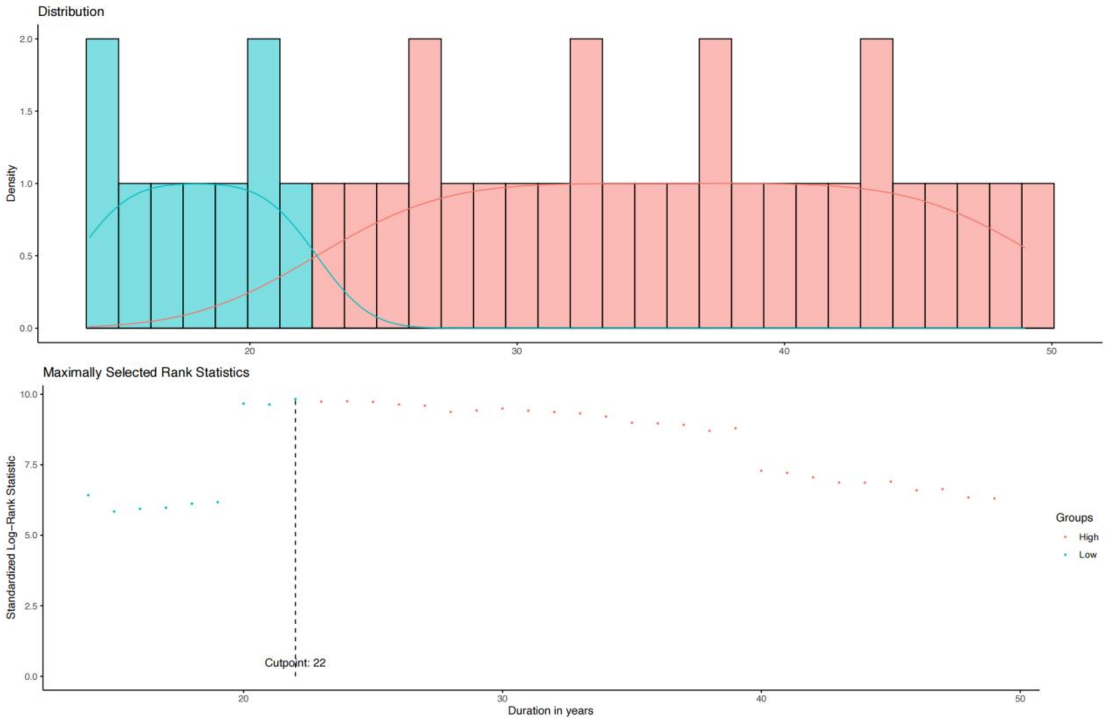
Distribution
Density
2.0
1.5
1.0
0.5
0.0
20
30
40
50
Maximally Selected Rank Statistics
Standardized Log-Rank Statistic
10.0
7.5
5.0
2.5
0.0
Cutpoint: 22
Duration in years
Groups
High
Low

Figure S6 : Cutoff value calculation among all smoking female participants.

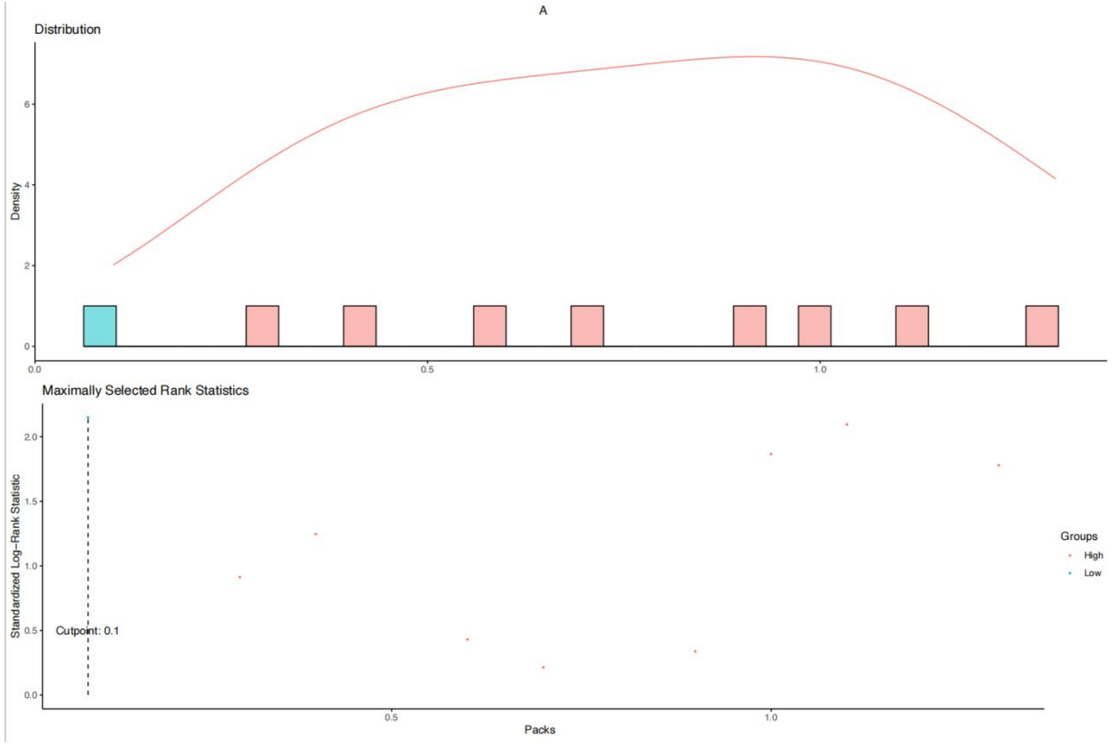

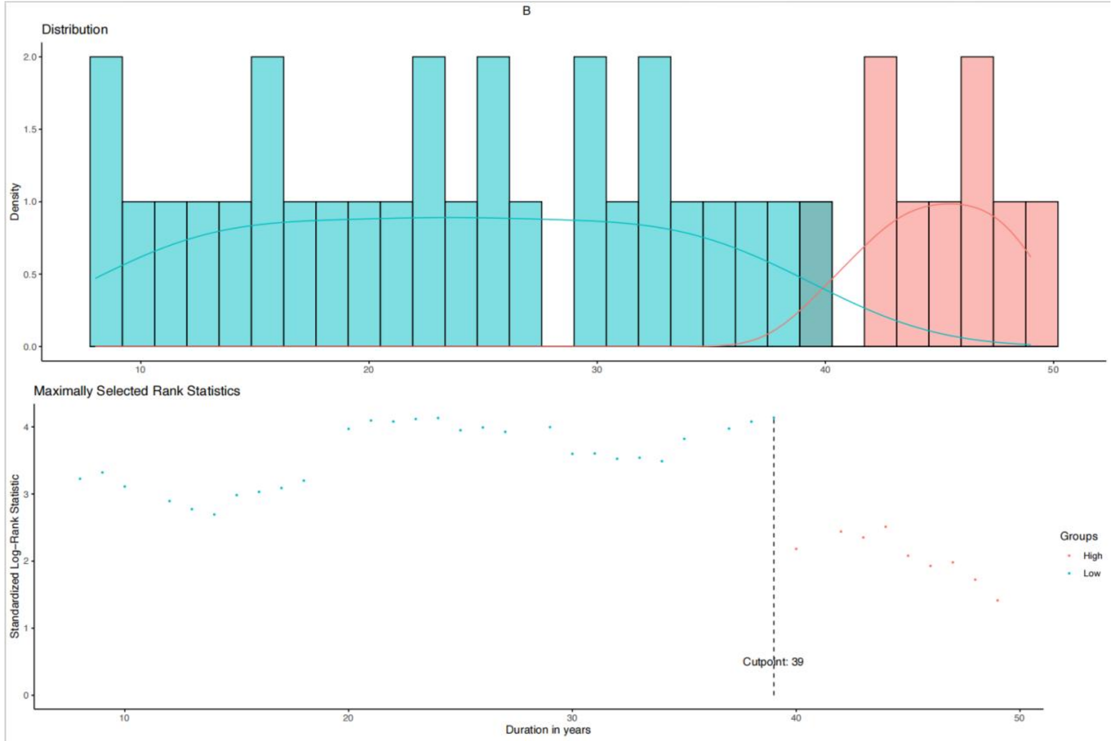
B
Distribution
Density
Maximally Selected Rank Statistics
Standardized Log-Rank Statistic
Cutpoint: 39
Groups
High
Low
Duration in years

Table S1 : The association between smoking status and nutritional condition stratified by sex.

Model*		OR (95% CI)	P value
All	E/T		
No smoking	9398/17598	1 [Reference]	
Smoking	7649/12390	1.15 (1.08,1.24)	<0.001
Male			
No smoking	2944/5037	1 [Reference]	
Smoking	7095/11445	1.13 (1.04,1.22)	0.003
Female			
No smoking	6454/12561	1 [Reference]	
Smoking	554/945	1.27 (1.09,1.49)	0.003

Model* was adjusted for sex, age, TNM, cancer types, surgery, chemotherapy, radiotherapy, occupation, BMI.

E/T: Malnutrition events/ Total number of patients with smoking status.

Table S2 : The association between smoking status and nutritional condition stratified by sex.

		Model 0		Model 1		Model 2	
All	Case	OR (95% CI)	P value	OR (95% CI)	P value	OR (95% CI)	P value
No smoking	17598	1 [Reference]		1 [Reference]		1 [Reference]	
Short duration in years and heavy packs	2187	1.22 (1.12,1.34)	<0.001	1.12 (1,1.25)	0.053	1.13 (1.01,1.27)	0.033
Long duration in years and light packs	2260	1.62 (1.48,1.78)	<0.001	1.3 (1.16,1.45)	<0.001	1.19 (1.06,1.34)	0.003
Male							
No smoking	5037	1 [Reference]		1 [Reference]		1 [Reference]	
Short duration in years and heavy packs	2044	1 (0.9,1.11)	0.98	1.1 (0.98,1.24)	0.115	1.09 (0.96,1.24)	0.173
Long duration in years and light packs	1988	1.35 (1.21,1.5)	<0.001	1.28 (1.13,1.45)	<0.001	1.17 (1.03,1.33)	0.017
Female							
No smoking	12561	1 [Reference]		1 [Reference]		1 [Reference]	
Short duration in years and heavy packs	143	1.17 (0.99,1.39)	0.074	1.2 (1,1.45)	0.057	1.25 (1.03,1.53)	0.023
Long duration in years and light packs	272	2.57 (1.13,6.58)	0.033	2.84 (1.01,10.09)	0.067	3.68 (1.26,13.46)	0.027

Model 0 was not adjusted for any covariates.

Model 1 was adjusted for sex, age, TNM, cancer types.

Model 2 was adjusted for sex, age, TNM, cancer types, surgery, chemotherapy, radiotherapy, occupation, BMI.

Figure S7 : Association between smoking and all-cause mortality in patients with solid tumours using a restricted cubic spline regression model among all smoking participants.

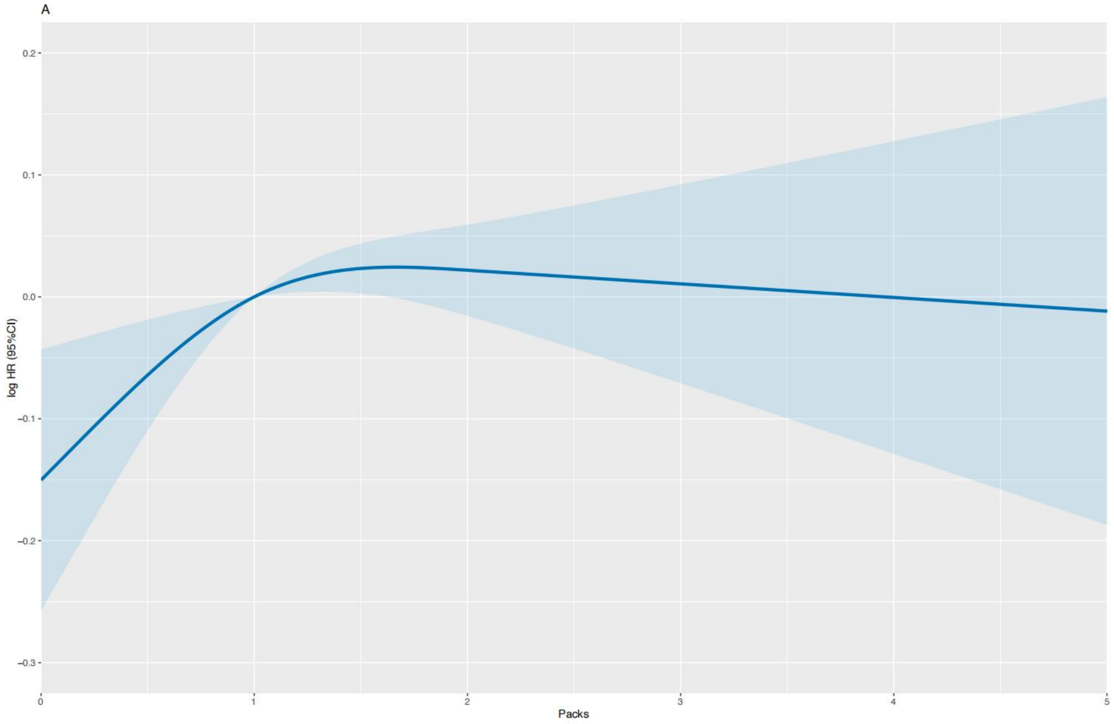

B

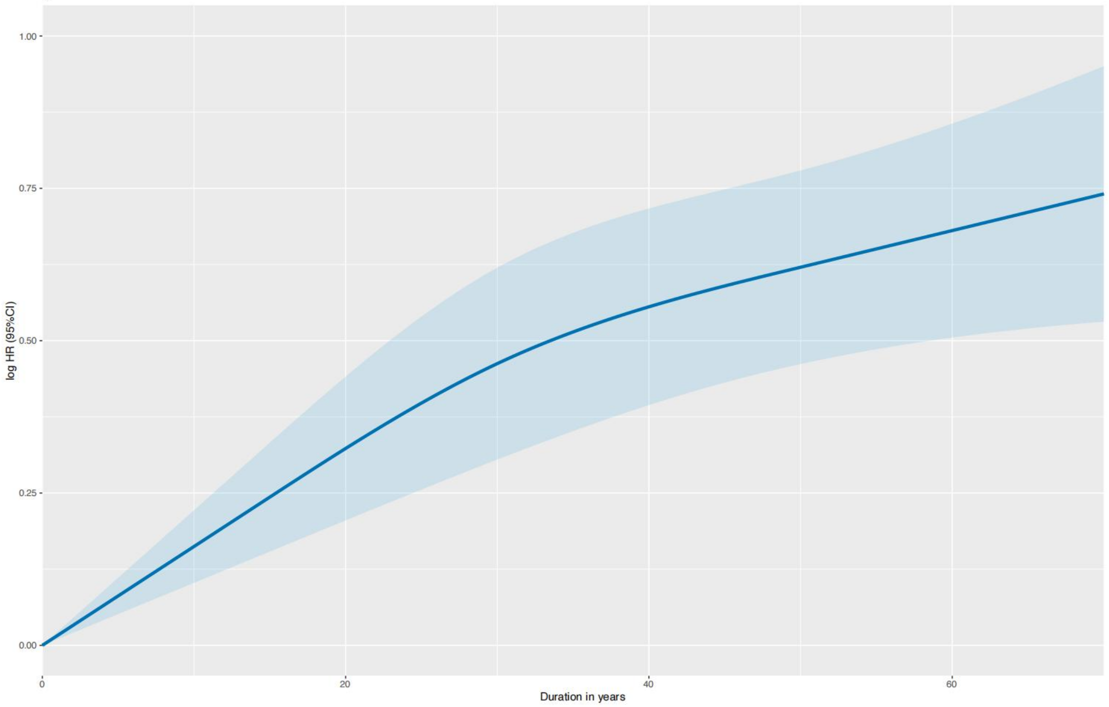

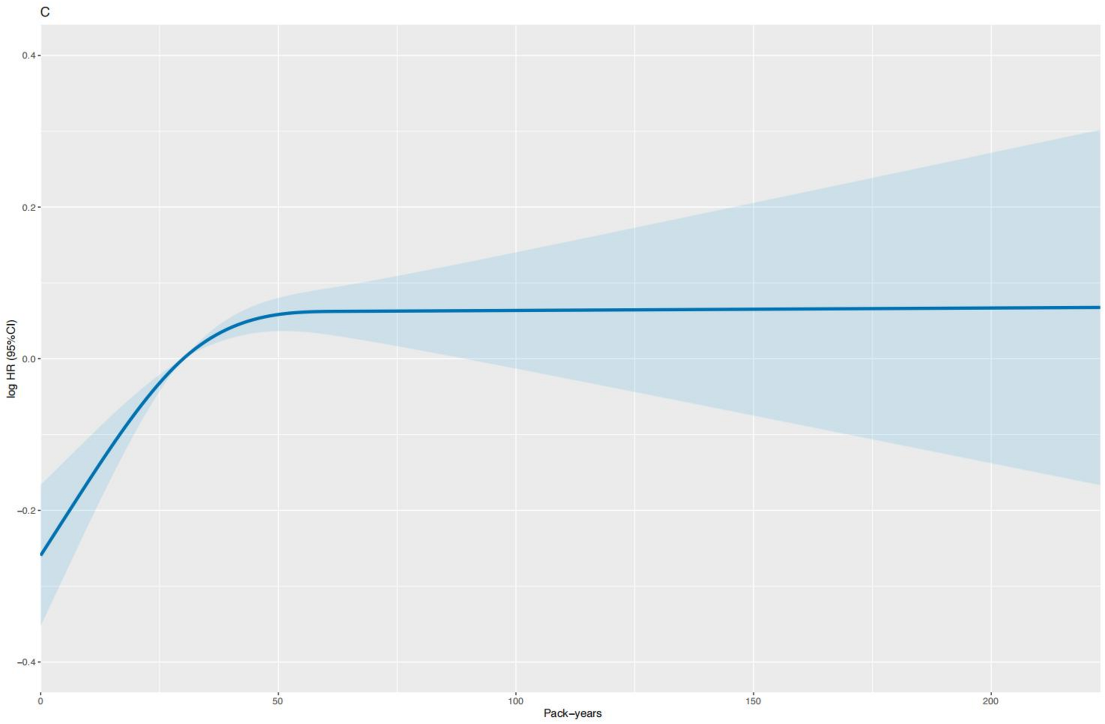

Model was adjusted for sex, age, TNM, cancer types, surgery, chemotherapy, radiotherapy, occupation, BMI.

Table S3 : Baseline Characteristics of Patients with Solid Tumours among smoking participants, Stratified by Smoking Duration Tertiles.

Characteristics	Patients, No. (%) T1 (N=1756)	Patients, No. (%) T2 (N=5676)	Patients, No. (%) T3 (N=4958)	P value
Sex				
Male	1525 (86.8)	5313 (93.6)	4607 (92.9)	<0.001

Female	231 (13.2)	363 (6.4)	351 (7.1)	
Age (mean (SD))	51.00 [42.00, 62.00]	56.00 [50.00, 63.00]	64.00 [59.00, 69.00]	<0.001
Packs (median [IQR])	1.00 [0.43, 1.00]	1.00 [0.57, 1.00]	1.00 [0.86, 1.43]	<0.001
Duration in years (median [IQR])	10.00 [8.00, 15.00]	30.00 [20.00, 30.00]	40.00 [40.00, 50.00]	<0.001
Pack-year (median [IQR])	7.14 [2.86, 13.00]	25.00 [17.14, 30.00]	40.00 [35.00, 57.14]	<0.001
Cancer types				
Nasopharyngeal Carcinoma	289 (16.5)	523 (9.2)	222 (4.5)	<0.001
Digestive System Cancers	910 (51.8)	2868 (50.5)	2211 (44.6)	
Lung Cancer	372 (21.2)	1937 (34.1)	2204 (44.5)	
Breast Cancer	70 (4.0)	81 (1.4)	56 (1.1)	
Genitourinary System Cancers	87 (5.0)	230 (4.1)	252 (5.1)	
Malignant Brain tumours	28 (1.6)	37 (0.7)	13 (0.3)	
TNM[#]				
I	131 (8.9)	483 (10.5)	455 (11.4)	0.26
II	288 (19.7)	920 (20.0)	799 (20.0)	
III	512 (34.9)	1578 (34.3)	1353 (33.9)	
IV	534 (36.5)	1619 (35.2)	1381 (34.6)	
Surgery				
No	1315 (74.9)	4300 (75.8)	3633 (73.3)	0.013
Yes	441 (25.1)	1376 (24.2)	1325 (26.7)	
Chemotherapy				
No	823 (46.9)	2639 (46.5)	2453 (49.5)	0.007
Yes	933 (53.1)	3037 (53.5)	2505 (50.5)	
Radiotherapy				

No	1464 (83.4)	4912 (86.5)	4423 (89.2)	<0.001
Yes	292 (16.6)	764 (13.5)	535 (10.8)	
BMI (mean (SD))	22.60 (3.40)	22.31 (3.35)	21.87 (3.30)	<0.001
PG-SGA				
<4	722 (41.1)	2295 (40.4)	1724 (34.8)	<0.001
≥4	1034 (58.9)	3381 (59.6)	3234 (65.2)	
COPD				
No	1748 (99.5)	5608 (98.8)	4820 (97.2)	<0.001
Yes	8 (0.5)	68 (1.2)	138 (2.8)	
Diabetes				
No	1623 (92.4)	5247 (92.4)	4519 (91.1)	0.035
Yes	133 (7.6)	429 (7.6)	439 (8.9)	
Hypertension				
No	1489 (84.8)	4791 (84.4)	3901 (78.7)	<0.001
Yes	267 (15.2)	885 (15.6)	1057 (21.3)	
Occupation				
Mental labor	288 (16.4)	802 (14.1)	392 (7.9)	<0.001
Physical labor	619 (35.3)	2292 (40.4)	1778 (35.9)	
Retirement and other	849 (48.3)	2582 (45.5)	2788 (56.2)	

T1:<20; T2:20-32; T3:≥32.

TNM[#]: 2337 data missing.

Digestive System Cancers included Esophagus cancer, Gastric cancer, Colorectal cancer, Liver cancer, Carcinoma of biliary tract, Pancreatic cancer.

Genitourinary System Cancers included Cervical cancer, Endometrial cancer, Ovarian cancer, Prostate cancer, Carcinoma of bladder.

Table S4 : The association of duration in years with all-cause mortality in smoking patients with solid tumours.

			Model 0		Model 1		Model 2	
All		Case	HR (95% CI)	P value	HR (95% CI)	P value	HR (95% CI)	P value
As continuous	(per 5 years)	12390	1.38 (1.3, 1.46)	<0.001	1.34 (1.24, 1.43)	<0.001	1.06 (1.04, 1.07)	<0.001
Low[a]		3731	1 [Reference]		1 [Reference]		1 [Reference]	
High[a]		8659	1.35 (1.27, 1.44)	<0.001	1.32 (1.22, 1.42)	<0.001	1.31 (1.21, 1.41)	<0.001
T1[b]		1756	1 [Reference]		1 [Reference]		1 [Reference]	
T2[b]		5676	1.16 (1.06, 1.27)	0.001	1.15 (1.03, 1.27)	0.009	1.14 (1.03, 1.26)	0.014
T3[b]		4958	1.48 (1.35, 1.62)	<0.001	1.42 (1.27, 1.59)	<0.001	1.40 (1.26, 1.57)	<0.001
P for trend				<0.001		<0.001		<0.001
Male								
As continuous	(per 5 years)	11445	1.06 (1.05, 1.08)	<0.001	1.06 (1.04, 1.07)	<0.001	1.32 (1.22, 1.42)	<0.001
Low[a]		3341	1 [Reference]		1 [Reference]		1 [Reference]	
High[a]		8104	1.33 (1.25, 1.42)	<0.001	1.31 (1.21, 1.42)	<0.001	1.3 (1.2, 1.41)	<0.001
T1[b]		1525	1 [Reference]		1 [Reference]		1 [Reference]	
T2[b]		5313	1.14 (1.03, 1.25)	0.009	1.14 (1.02, 1.27)	0.016	1.13 (1.02, 1.26)	0.023
T3[b]		4607	1.44 (1.31, 1.58)	<0.001	1.41 (1.25, 1.58)	<0.001	1.39 (1.24, 1.56)	<0.001
P for trend				<0.001		<0.001		<0.001
Female								
As continuous	(per 5 years)	945	1.09 (1.05, 1.13)	<0.001	1.07 (1.03, 1.12)	0.002	1.36 (1.07, 1.72)	0.011
Low[c]		392	1 [Reference]		1 [Reference]		1 [Reference]	
High[c]		553	1.6 (1.27, 2.02)	<0.001	1.49 (1.14, 1.95)	0.003	1.42 (1.07, 1.86)	0.013
T1[b]		231	1 [Reference]		1 [Reference]		1 [Reference]	
T2[b]		363	1.32 (0.97, 1.79)	0.074	1.13 (0.81, 1.58)	0.483	1.09 (0.78, 1.53)	0.604

T3[b]	351	1.8 (1.34, 2.43)	<0.001	1.69 (1.19, 2.4)	0.003	1.58 (1.1, 2.27)	0.013
P for trend			<0.001		0.002		0.009

Model 0 was not adjusted for any covariates.

Model 1 was adjusted for sex, age, TNM, cancer types.

Model 2 was adjusted for sex, age, TNM, cancer types, surgery, chemotherapy, radiotherapy, occupation, BMI.

Low[a]:<22; High[a]:≥22.

T1[b]:<20; T2[b]:20-32; T3[b]:≥32.

Low[C]:<39; High[C]:≥39.

Figure S8 : Association between smoking and all-cause mortality in patients with solid tumours using a restricted cubic spline regression model among all malnutrition participants.

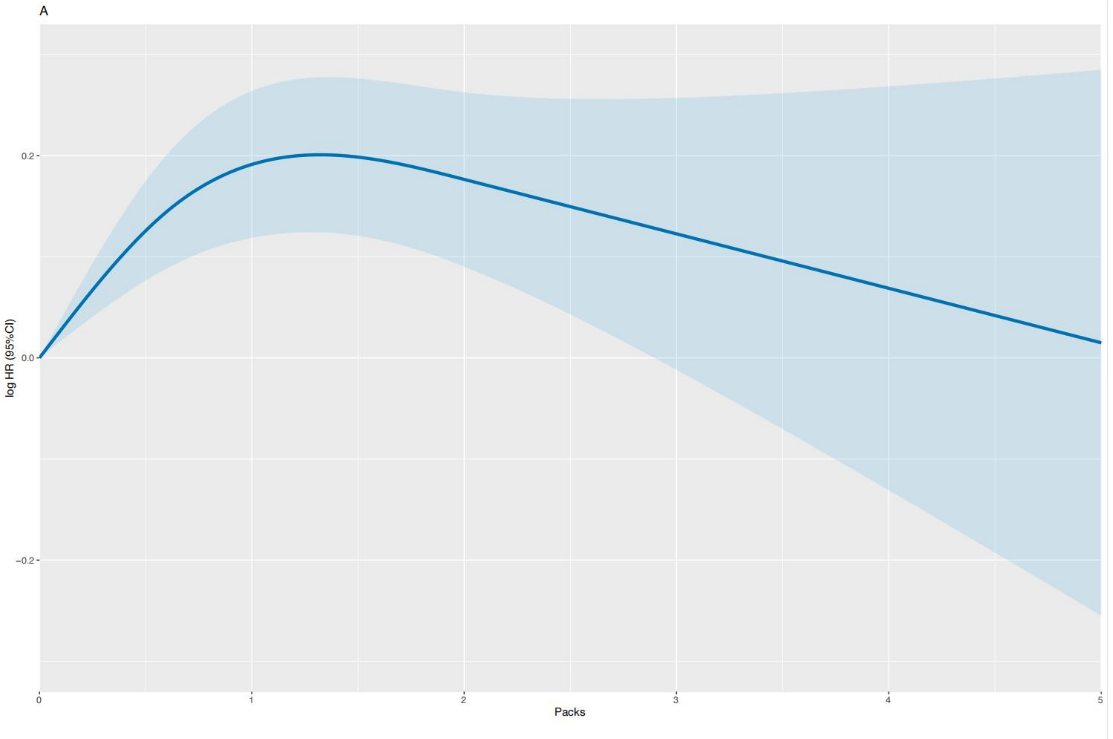

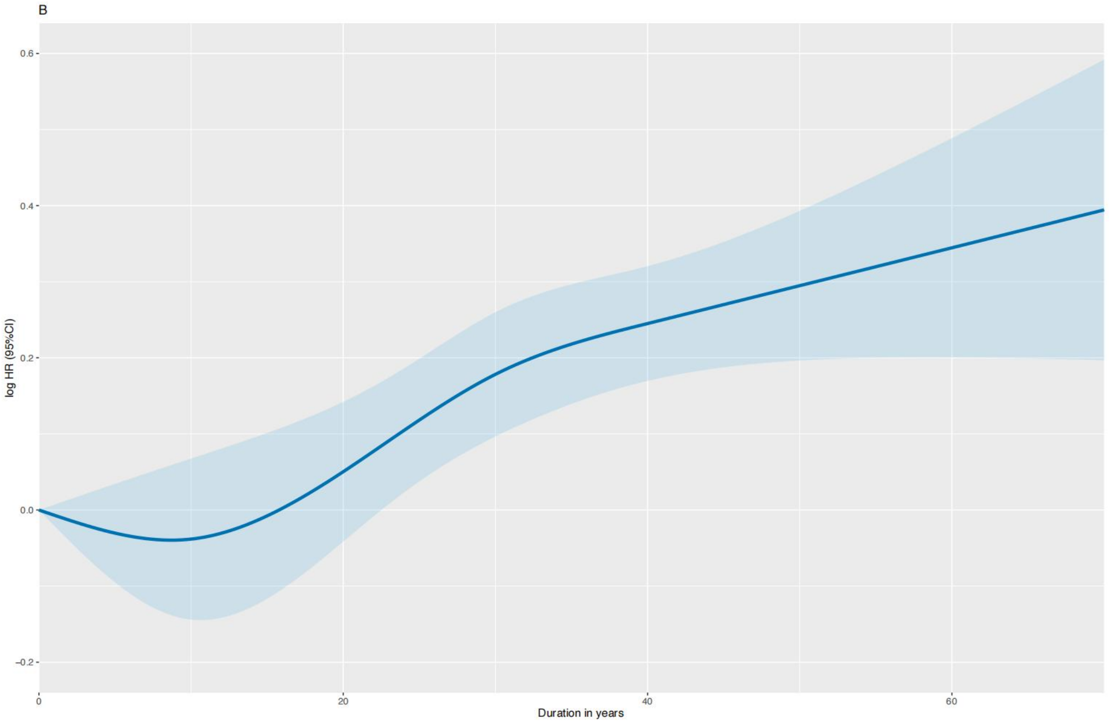
B
0.6
0.4
0.2
0.0
−0.2
log HR (95%CI)
0
20
40
60
Duration in years

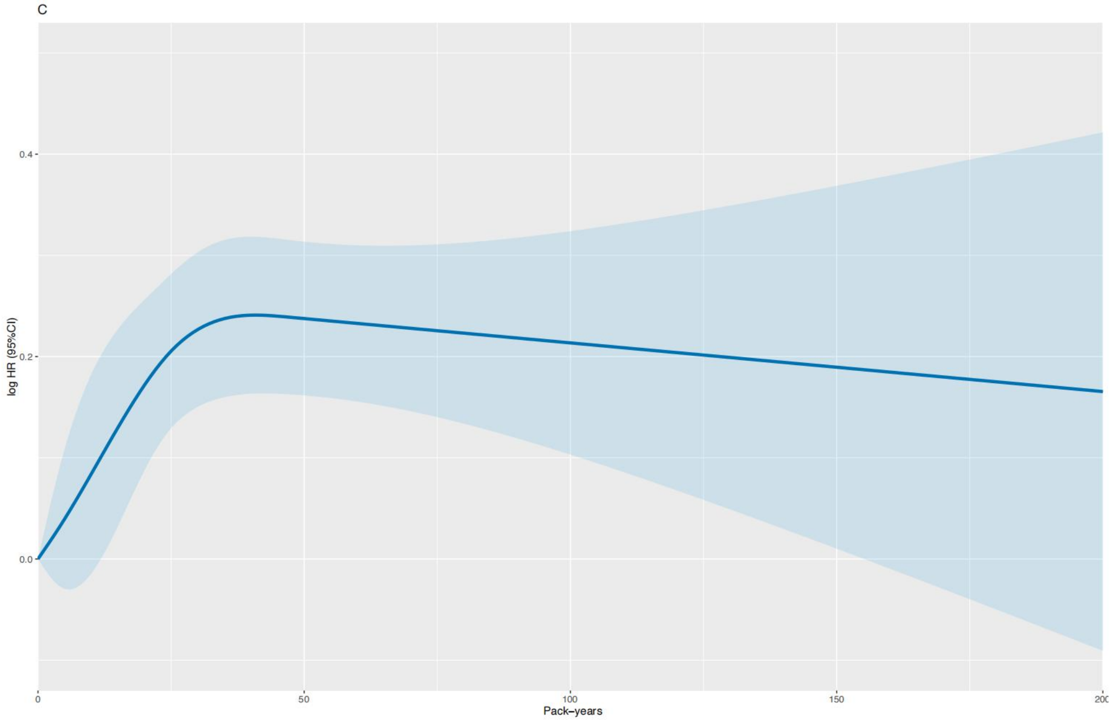

Model was adjusted for sex, age, TNM, cancer types, surgery, chemotherapy, radiotherapy, occupation, BMI.

Figure S9 : Cutoff value calculation among all malnutrition participants.

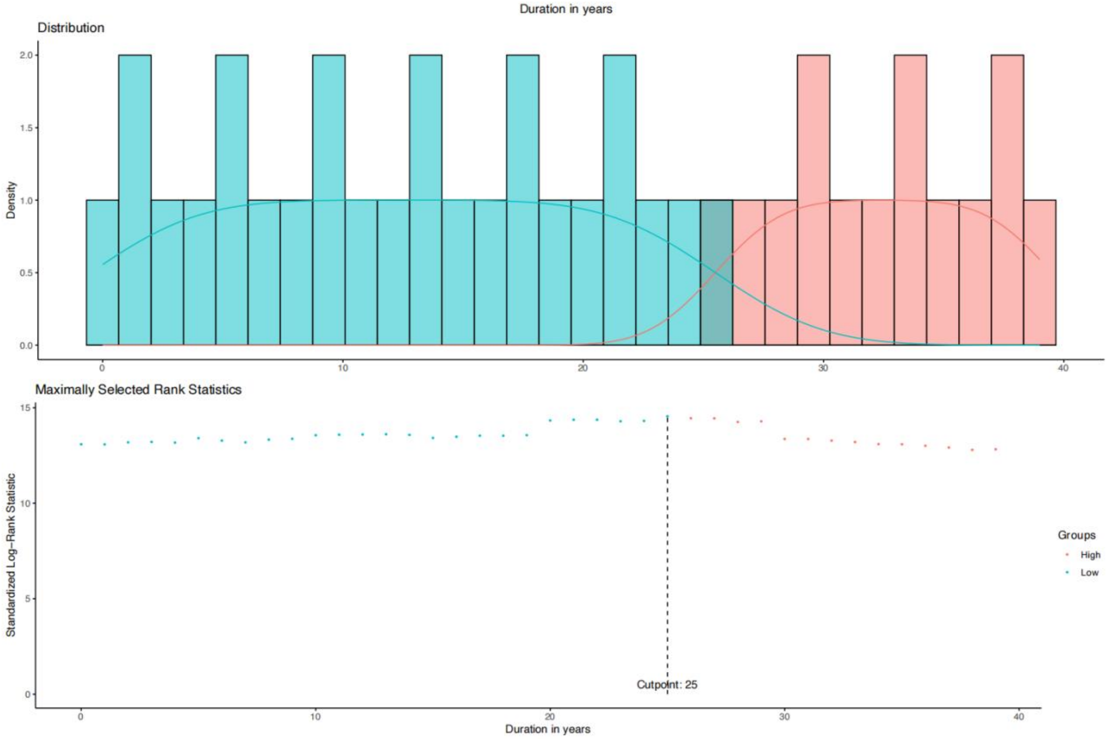

Table S5 : Baseline Characteristics of Patients with Solid Tumours among malnutrition participants, Stratified by Smoking Duration Tertiles.

Characteristics	Patients, No. (%)<br>T1 (N=9379)	Patients, No. (%)<br>T2 (N=2209)	Patients, No. (%)<br>T3 (N=5459)	P value
Sex				
Male	2924 (31.2)	1991 (90.1)	5124 (93.9)	<0.001
Female	6455 (68.8)	218 (9.9)	335 (6.1)	

Age (mean (SD))	57.14 (12.40)	54.96 (12.53)	62.25 (8.87)	<0.001
Smoking				
No	9371 (99.9)	16 (0.7)	11 (0.2)	<0.001
Yes	8 (0.1)	2193 (99.3)	5448 (99.8)	
Packs (median [IQR])	0.00 [0.00, 0.00]	1.00 [0.43, 1.00]	1.00 [0.71, 1.43]	<0.001
Duration in years (median [IQR])	0.00 [0.00, 0.00]	20.00 [10.00, 20.00]	40.00 [30.00, 40.00]	<0.001
Pack-year (median [IQR])	0.00 [0.00, 0.00]	12.00 [5.71, 20.00]	37.14 [25.00, 50.00]	<0.001
Cancer types				
Nasopharyngeal Carcinoma	372 (4.0)	208 (9.4)	204 (3.7)	<0.001
Digestive System Cancers	4866 (51.9)	1347 (61.0)	3039 (55.7)	
Lung Cancer	1708 (18.2)	523 (23.7)	2004 (36.7)	
Breast Cancer	1069 (11.4)	42 (1.9)	49 (0.9)	
Genitourinary System Cancers	1280 (13.6)	66 (3.0)	143 (2.6)	
Malignant Brain tumours	84 (0.9)	23 (1.0)	20 (0.4)	
TNM[#]				
I	970 (12.7)	161 (8.9)	427 (9.7)	<0.001
II	1765 (23.1)	347 (19.1)	866 (19.7)	
III	2437 (31.8)	646 (35.6)	1465 (33.3)	
IV	2482 (32.4)	662 (36.5)	1638 (37.3)	
Surgery				
No	6809 (72.6)	1572 (71.2)	3984 (73.0)	0.266
Yes	2570 (27.4)	637 (28.8)	1475 (27.0)	
Chemotherapy				
No	4716 (50.3)	1117 (50.6)	2769 (50.7)	0.869

Yes	4663 (49.7)	1092 (49.4)	2690 (49.3)	
Radiotherapy				
No	8432 (89.9)	1909 (86.4)	4832 (88.5)	<0.001
Yes	947 (10.1)	300 (13.6)	627 (11.5)	
BMI (mean (SD))	21.87 (3.48)	21.75 (3.33)	21.36 (3.24)	<0.001
COPD				
No	9331 (99.5)	2193 (99.3)	5339 (97.8)	<0.001
Yes	48 (0.5)	16 (0.7)	120 (2.2)	
Diabetes				
No	8605 (91.7)	2049 (92.8)	5021 (92.0)	0.291
Yes	774 (8.3)	160 (7.2)	438 (8.0)	
Hypertension				
No	7619 (81.2)	1873 (84.8)	4405 (80.7)	<0.001
Yes	1760 (18.8)	336 (15.2)	1054 (19.3)	
Occupation				
Mental labor	1085 (11.6)	316 (14.3)	545 (10.0)	<0.001
Physical labor	3359 (35.8)	858 (38.8)	2103 (38.5)	
Retirement and other	4935 (52.6)	1035 (46.9)	2811 (51.5)	

T1:0; T2:0-20; T3:≥20.

TNM#: 3181 data missing.

Digestive System Cancers included Esophagus cancer, Gastric cancer, Colorectal cancer, Liver cancer, Carcinoma of biliary tract, Pancreatic cancer.

Genitourinary System Cancers included Cervical cancer, Endometrial cancer, Ovarian cancer, Prostate cancer, Carcinoma of bladder.

Table S6 : The association of duration in years with all-cause mortality in malnutrition patients with solid tumours.

	Model 0	Model 1	Model 2

All		Case	HR (95% CI)	P value	HR (95% CI)	P value	HR (95% CI)	P value
As continuous	(per 10 years)	17047	1.11 (1.1, 1.13)	<0.001	1.06 (1.04, 1.08)	<0.001	1.86 (1.55, 2.23)	<0.001
Low[a]		11653	1 [Reference]		1 [Reference]		1 [Reference]	
High[a]		5394	1.46 (1.39, 1.54)	<0.001	1.24 (1.16, 1.32)	<0.001	1.24 (1.16, 1.32)	<0.001
T1[b]		9379	1 [Reference]		1 [Reference]		1 [Reference]	
T2[b]		2209	1.14 (1.06, 1.23)	0.001	1.02 (0.92, 1.12)	0.741	1.02 (0.93, 1.12)	0.687
T3[b]		5459	1.5 (1.42, 1.58)	<0.001	1.25 (1.16, 1.34)	<0.001	1.25 (1.16, 1.35)	<0.001
P for trend				<0.001		<0.001		<0.001
Male								
As continuous	(per 10 years)	10039	1.08 (1.06, 1.1)	<0.001	1.06 (1.04, 1.08)	<0.001	1.74 (1.43, 2.12)	<0.001
Low[a]		4976	1 [Reference]		1 [Reference]		1 [Reference]	
High[a]		5063	1.29 (1.22, 1.37)	<0.001	1.21 (1.13, 1.3)	<0.001	1.21 (1.13, 1.3)	<0.001
T1[b]		2924	1 [Reference]		1 [Reference]		1 [Reference]	
T2[b]		1991	0.98 (0.89, 1.07)	0.601	0.98 (0.88, 1.08)	0.66	0.98 (0.88, 1.09)	0.702
T3[b]		5124	1.28 (1.19, 1.37)	<0.001	1.2 (1.11, 1.3)	<0.001	1.21 (1.11, 1.31)	<0.001
P for trend				<0.001		<0.001		<0.001
Female								
As continuous	(per 10 years)	7008	1.12 (1.08, 1.17)	<0.001	1.11 (1.06, 1.16)	<0.001	2.88 (1.8, 4.59)	<0.001
Low[a]		6677	1 [Reference]		1 [Reference]		1 [Reference]	
High[a]		331	1.53 (1.29, 1.83)	<0.001	1.46 (1.19, 1.78)	<0.001	1.46 (1.2, 1.79)	<0.001
T1[b]		6455	1 [Reference]		1 [Reference]		1 [Reference]	
T2[b]		218	1.24 (0.99, 1.55)	0.067	1.18 (0.92, 1.51)	0.187	1.19 (0.93, 1.52)	0.167
T3[b]		335	1.56 (1.31, 1.85)	<0.001	1.48 (1.21, 1.81)	<0.001	1.49 (1.22, 1.82)	<0.001
P for trend				<0.001		<0.001		<0.001

Model 0 was not adjusted for any covariates.
Model 1 was adjusted for sex, age, TNM, cancer types.
Model 2 was adjusted for sex, age, TNM, cancer types, surgery, chemotherapy, radiotherapy, occupation, BMI.
Low[a]:<25; High[a]:≥25.
T1[b]:0; T2[b]:0-20; T3[b]:≥20.

Figure S10 : Flow Chart.

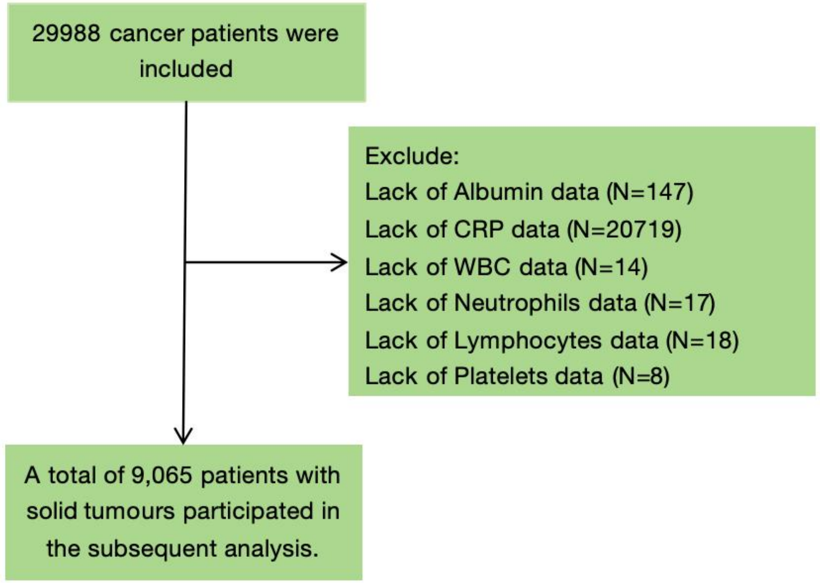

Figure S11 : Cutoff value calculation among all smoking participants.

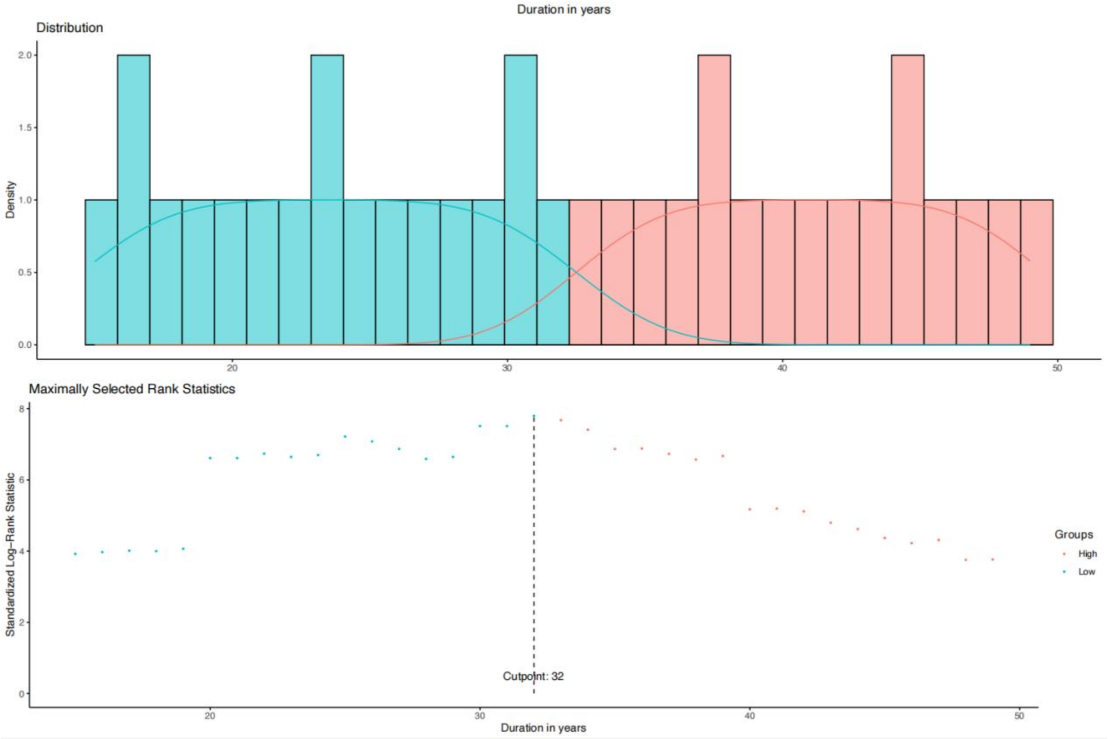

Table S7 : Baseline Characteristics of Patients with Solid Tumours, Stratified by Smoking Duration Cutoff Values.

Characteristics	Duration in years			P value
	Patients, No. (%)<br>0 (N=4697)	Patients, No. (%)<br><32 (N=2467)	Patients, No. (%)<br>≥32 (N=1901)	
Sex				
Male	1538 (32.7)	2205 (89.4)	1730 (91.0)	<0.001

Female	3159 (67.3)	262 (10.6)	171 (9.0)	
Age (mean (SD))	57.38 (11.94)	56.62 (10.83)	64.10 (7.48)	<0.001
Smoking				
No	4697 (100.0)	12 (0.5)	8 (0.4)	<0.001
Yes	0 (0.0)	2455 (99.5)	1893 (99.6)	
Packs (median [IQR])	0.00 [0.00, 0.00]	1.00 [0.57, 0.86]	1.00 [1.00, 1.29]	<0.001
Duration in years (median [IQR])	0.00 [0.00, 0.00]	25.00 [20.00, 30.00]	40.00 [40.00, 50.00]	<0.001
Pack-year (median [IQR])	0.00 [0.00, 0.00]	20.00 [10.00, 30.00]	40.00 [34.29, 57.14]	<0.001
Cancer types				
Nasopharyngeal Carcinoma	90 (1.9)	73 (3.0)	33 (1.7)	<0.001
Digestive System Cancers	2370 (50.5)	1335 (54.1)	767 (40.3)	
Lung Cancer	1150 (24.5)	898 (36.4)	971 (51.1)	
Breast Cancer	642 (13.7)	32 (1.3)	13 (0.7)	
Genitourinary System Cancers	430 (9.2)	123 (5.0)	117 (6.2)	
Malignant Brain tumours	15 (0.3)	6 (0.2)	0 (0.0)	
TNM[#]				
I	547 (13.0)	231 (10.7)	174 (10.9)	<0.001
II	870 (20.6)	394 (18.3)	268 (16.8)	
III	1367 (32.4)	734 (34.2)	540 (33.8)	
IV	1430 (33.9)	790 (36.8)	616 (38.5)	
Surgery				
No	3714 (79.1)	1893 (76.7)	1496 (78.7)	0.068
Yes	983 (20.9)	574 (23.3)	405 (21.3)	
Chemotherapy				

No	2207 (47.0)	1098 (44.5)	853 (44.9)	0.083
Yes	2490 (53.0)	1369 (55.5)	1048 (55.1)	
Radiotherapy				
No	4445 (94.6)	2327 (94.3)	1786 (94.0)	0.537
Yes	252 (5.4)	140 (5.7)	115 (6.0)	
BMI (mean (SD))	22.67 (3.48)	22.53 (3.41)	21.99 (3.27)	<0.001
COPD				
No	4662 (99.3)	2429 (98.5)	1825 (96.0)	<0.001
Yes	35 (0.7)	38 (1.5)	76 (4.0)	
Diabetes				
No	4219 (89.8)	2245 (91.0)	1694 (89.1)	0.101
Yes	478 (10.2)	222 (9.0)	207 (10.9)	
Hypertension				
No	3767 (80.2)	2046 (82.9)	1487 (78.2)	<0.001
Yes	930 (19.8)	421 (17.1)	414 (21.8)	
Occupation				
Mental labor	611 (13.0)	357 (14.5)	147 (7.7)	<0.001
Physical labor	1599 (34.0)	1017 (41.2)	713 (37.5)	
Retirement and other	2487 (52.9)	1093 (44.3)	1041 (54.8)	
Total Protein (g/L)	68.40 [63.60, 73.10]	67.70 [63.10, 72.20]	67.70 [63.10, 72.00]	<0.001
Creatinine (μmol/L)	61.55 [52.10, 74.00]	70.35 [60.80, 82.00]	70.70 [60.60, 83.00]	<0.001
Albumin (g/L)	39.50 [35.70, 42.50]	39.10 [35.60, 42.30]	38.30 [34.60, 41.50]	<0.001
Blood Urea Nitrogen (mmol/L)	4.91 [3.91, 6.09]	5.30 [4.26, 6.57]	5.55 [4.43, 6.81]	<0.001
Total Bilirubin (μmol/L)	10.50 [7.65, 14.50]	10.90 [8.20, 15.05]	10.55 [7.88, 14.40]	0.614

CRP (mg/L)	3.50 [1.79, 13.60]	5.00 [2.60, 21.20]	6.00 [2.87, 24.90]	<0.001
Hemoglobin (g/L)	121.00 [106.00, 134.00]	130.00 [114.00, 143.00]	129.00 [113.00, 142.00]	<0.001
WBC ($\times10^9$/L)	5.70 [4.47, 7.31]	6.20 [4.80, 7.97]	6.47 [5.10, 8.40]	<0.001
Neutrophil ($\times10^9$/L)	3.50 [2.48, 5.10]	3.91 [2.70, 5.50]	4.07 [2.86, 5.83]	<0.001
Lymphocytes ($\times10^9$/L)	1.40 [1.00, 1.81]	1.47 [1.06, 1.90]	1.51 [1.09, 2.00]	<0.001
Platelets ($\times10^9$/L)	219.00 [168.00, 282.00]	216.00 [165.00, 280.00]	222.00 [170.00, 281.00]	0.421

TNM#: 1104 data missing.

Digestive System Cancers included Esophagus cancer, Gastric cancer, Colorectal cancer, Liver cancer, Carcinoma of biliary tract, Pancreatic cancer.

Genitourinary System Cancers included Cervical cancer, Endometrial cancer, Ovarian cancer, Prostate cancer, Carcinoma of bladder.

Table S8 : Baseline Characteristics of Patients with Solid Tumours, Stratified by PG-SGA scores Values.

Characteristics	PG-SGA				P value
	Patients, No. (%) 0-1 (N=1443)	Patients, No. (%) 2-3 (N=2036)	Patients, No. (%) 4-8 (N=3101)	Patients, No. (%) ≥9 (N=2485)	
Sex					
Male	753 (52.2)	1228 (60.3)	1937 (62.5)	1555 (62.6)	<0.001
Female	690 (47.8)	808 (39.7)	1164 (37.5)	930 (37.4)	
Age (mean (SD))	52.41 (8.66)	60.10 (10.98)	58.43 (11.19)	61.11 (11.38)	<0.001
Smoking					
No	809 (56.1)	1057 (51.9)	1577 (50.9)	1274 (51.3)	0.008
Yes	634 (43.9)	979 (48.1)	1524 (49.1)	1211 (48.7)	
Packs (median [IQR])	0.00 [0.00, 1.00]	0.00 [0.00, 1.00]	0.00 [0.00, 1.00]	0.00 [0.00, 1.00]	0.002
Duration in years (median [IQR])	0.00 [0.00, 30.00]	0.00 [0.00, 30.00]	0.00 [0.00, 30.00]	0.00 [0.00, 30.00]	<0.001
Pack-year (median [IQR])	0.00 [0.00, 22.43]	0.00 [0.00, 30.00]	0.00 [0.00, 30.00]	0.00 [0.00, 30.00]	<0.001
Cancer types					

Nasopharyngeal Carcinoma	74 (5.1)	42 (2.1)	45 (1.5)	35 (1.4)	<0.001
Digestive System Cancers	396 (27.4)	776 (38.1)	1724 (55.6)	1576 (63.4)	
Lung Cancer	593 (41.1)	788 (38.7)	985 (31.8)	653 (26.3)	
Breast Cancer	235 (16.3)	200 (9.8)	174 (5.6)	78 (3.1)	
Genitourinary System Cancers	137 (9.5)	227 (11.1)	164 (5.3)	142 (5.7)	
Malignant Brain tumours	8 (0.6)	3 (0.1)	9 (0.3)	1 (0.0)	
TNM[#]					
I	217 (17.3)	263 (14.9)	306 (11.1)	166 (7.6)	<0.001
II	288 (23.0)	373 (21.1)	521 (18.9)	350 (16.0)	
III	378 (30.2)	592 (33.4)	980 (35.6)	691 (31.6)	
IV	370 (29.5)	543 (30.7)	943 (34.3)	980 (44.8)	
Surgery					
No	1240 (85.9)	1588 (78.0)	2389 (77.0)	1886 (75.9)	<0.001
Yes	203 (14.1)	448 (22.0)	712 (23.0)	599 (24.1)	
Chemotherapy					
No	604 (41.9)	926 (45.5)	1328 (42.8)	1300 (52.3)	<0.001
Yes	839 (58.1)	1110 (54.5)	1773 (57.2)	1185 (47.7)	
Radiotherapy					
No	1330 (92.2)	1929 (94.7)	2939 (94.8)	2360 (95.0)	0.001
Yes	113 (7.8)	107 (5.3)	162 (5.2)	125 (5.0)	
BMI (mean (SD))	24.12 (3.14)	23.37 (3.24)	22.38 (3.27)	20.95 (3.27)	<0.001
COPD					
No	1432 (99.2)	1993 (97.9)	3051 (98.4)	2440 (98.2)	0.017
Yes	11 (0.8)	43 (2.1)	50 (1.6)	45 (1.8)	

Diabetes					
No	1326 (91.9)	1813 (89.0)	2790 (90.0)	2229 (89.7)	0.045
Yes	117 (8.1)	223 (11.0)	311 (10.0)	256 (10.3)	
Hypertension					
No	1234 (85.5)	1614 (79.3)	2478 (79.9)	1974 (79.4)	<0.001
Yes	209 (14.5)	422 (20.7)	623 (20.1)	511 (20.6)	
Occupation					
Mental labor	280 (19.4)	224 (11.0)	358 (11.5)	253 (10.2)	<0.001
Physical labor	511 (35.4)	663 (32.6)	1159 (37.4)	996 (40.1)	
Retirement and other	652 (45.2)	1149 (56.4)	1584 (51.1)	1236 (49.7)	
Total Protein (g/L)	69.30 [65.50, 73.55]	69.40 [64.97, 73.70]	67.80 [63.10, 72.40]	66.30 [60.90, 71.30]	<0.001
Creatinine (μmol/L)	63.40 [55.00, 74.30]	67.15 [57.20, 79.32]	66.95 [56.00, 79.00]	66.00 [54.70, 79.40]	<0.001
Albumin (g/L)	41.00 [38.10, 43.70]	40.50 [37.27, 43.00]	39.00 [35.50, 42.00]	36.50 [32.70, 40.20]	<0.001
Blood Urea Nitrogen (mmol/L)	5.09 [4.19, 6.18]	5.26 [4.32, 6.47]	5.12 [4.08, 6.35]	5.07 [3.89, 6.56]	<0.001
Total Bilirubin (μmol/L)	10.60 [8.20, 14.30]	10.70 [7.93, 14.60]	10.55 [7.80, 14.38]	10.80 [7.60, 15.40]	<0.001
CRP (mg/L)	3.11 [1.23, 6.28]	3.23 [1.40, 9.62]	4.83 [2.60, 18.20]	10.00 [3.20, 40.70]	<0.001
Hemoglobin (g/L)	132.00 [120.00, 143.00]	129.00 [116.00, 142.00]	125.00 [110.00, 138.00]	116.00 [98.00, 131.00]	<0.001
WBC ($\times 10^9$/L)	5.70 [4.51, 7.24]	5.88 [4.63, 7.40]	5.95 [4.62, 7.60]	6.39 [4.84, 8.71]	<0.001
Neutrophil ($\times 10^9$/L)	3.40 [2.44, 4.72]	3.50 [2.54, 4.87]	3.70 [2.55, 5.24]	4.37 [2.88, 6.50]	<0.001
Lymphocytes ($\times 10^9$/L)	1.55 [1.16, 2.00]	1.52 [1.11, 2.00]	1.45 [1.06, 1.86]	1.27 [0.87, 1.72]	<0.001
Platelets ($\times 10^9$/L)	213.00 [167.00, 263.00]	212.00 [167.00, 266.00]	222.00 [168.00, 285.00]	227.00 [168.00, 301.00]	<0.001

TNM[#]: 1104 data missing.

Digestive System Cancers included Esophagus cancer, Gastric cancer, Colorectal cancer, Liver cancer, Carcinoma of biliary tract, Pancreatic cancer.

Genitourinary System Cancers included Cervical cancer, Endometrial cancer, Ovarian cancer, Prostate cancer, Carcinoma of bladder.

Table S9 : Univariate and Multivariate Cox Regression Analyses of Hematological Indicators in Smoking Populations.

Characteristics	Univariable HR (95% CI, P)	Multivariable HR (95% CI, P)
Total Protein (g/L)	0.98 (0.87-1.10, p=0.720)	
Albumin (g/L)	0.83 (0.71-0.96, p=0.014)	0.96 (0.87-1.07, p=0.497)
CRP (mg/L)	1.06 (1.04-1.09, p<0.001)	1.04 (1.02-1.06, p=0.001)
WBC ($\times 10^9$/L)	1.60 (1.33-1.93, p<0.001)	1.28 (1.03-1.61, p=0.028)
Neutrophil ($\times 10^9$/L)	1.15 (1.03-1.27, p=0.011)	1.03 (0.90-1.18, p=0.639)
Lymphocytes ($\times 10^9$/L)	0.95 (0.62-1.47, p=0.832)	
Platelets ($\times 10^9$/L)	1.02 (1.02-1.03, p<0.001)	1.02 (1.01-1.03, p<0.001)
Creatinine (μmol/L)	0.98 (0.94-1.02, p=0.275)	
Blood Urea Nitrogen (mmol/L)	1.01 (0.97-1.06, p=0.561)	
Total Bilirubin (μmol/L)	1.08 (1.04-1.13, p<0.001)	1.08 (1.03-1.13, p=0.001)
Hemoglobin (g/L)	0.96 (0.93-0.99, p=0.019)	0.99 (0.96-1.03, p=0.573)

Table S10 : Univariate and Multivariate Cox Regression Analyses of Hematological Indicators in malnutrition Populations.

Characteristics	Univariable HR (95% CI, P)	Multivariable HR (95% CI, P)
Total Protein (g/L)	0.95 (0.86-1.06, p=0.351)	
Albumin (g/L)	0.71 (0.62-0.82, p<0.001)	0.87 (0.75-1.00, p=0.047)
CRP (mg/L)	1.05 (1.03-1.07, p<0.001)	1.02 (1.00-1.04, p=0.024)
WBC ($\times 10^9$/L)	1.67 (1.44-1.93, p<0.001)	1.41 (1.17-1.69, p<0.001)
Neutrophil ($\times 10^9$/L)	1.10 (1.01-1.20, p=0.025)	1.03 (0.92-1.15, p=0.581)
Lymphocytes ($\times 10^9$/L)	0.75 (0.51-1.12, p=0.158)	

Platelets (×10^9/L)	1.02 (1.01-1.03, p<0.001)	1.01 (1.00-1.02, p=0.002)
Creatinine (μmol/L)	1.01 (1.00-1.03, p=0.147)	
Blood Urea Nitrogen (mmol/L)	1.00 (0.96-1.04, p=0.950)	
Total Bilirubin (μmol/L)	1.07 (1.04-1.10, p<0.001)	1.07 (1.03-1.10, p<0.001)
Hemoglobin (g/L)	1.02 (0.99-1.05, p=0.217)	

Table S11 : Sensitivity Analysis of the Association Between Smoking Duration and All-Cause Mortality in Smoking Patients.

Excluding patients with follow-up less than 1 year	HR (95% CI)	P value	HR (95% CI)	P value	HR (95% CI)	P value
As continuous （per 5 years)	1.06 (1.04, 1.07)	<0.001	1.05 (1.03, 1.07)	<0.001	1.27 (1.17, 1.37)	<0.001
Low[a]	1 [Reference]		1 [Reference]		1 [Reference]	
High[a]	1.29 (1.2, 1.38)	<0.001	1.25 (1.15, 1.35)	<0.001	1.24 (1.14, 1.34)	<0.001
T1[b]	1 [Reference]		1 [Reference]		1 [Reference]	
T2[b]	1.09 (0.99, 1.2)	0.071	1.08 (0.96, 1.2)	0.187	1.07 (0.96, 1.19)	0.225
T3[b]	1.37 (1.24, 1.51)	<0.001	1.31 (1.17, 1.48)	<0.001	1.3 (1.15, 1.46)	<0.001
P for trend		<0.001		<0.001		<0.001
Excluding patients with BMI>35kg/m^2						
As continuous （per 5 years)	1.07 (1.05, 1.08)	<0.001	1.06 (1.04, 1.07)	<0.001	1.32 (1.23, 1.42)	<0.001
Low[a]	1 [Reference]		1 [Reference]		1 [Reference]	
High[a]	1.35 (1.27, 1.44)	<0.001	1.32 (1.22, 1.42)	<0.001	1.31 (1.21, 1.41)	<0.001
T1[b]	1 [Reference]		1 [Reference]		1 [Reference]	
T2[b]	1.16 (1.06, 1.27)	0.001	1.15 (1.03, 1.27)	0.009	1.14 (1.03, 1.26)	0.015
T3[b]	1.48 (1.35, 1.62)	<0.001	1.42 (1.27, 1.59)	<0.001	1.4 (1.26, 1.56)	<0.001

P for trend		<0.001		<0.001		<0.001
Additionally adjusted for time-varying covariates						
As continuous （per 5 years)	1.07 (1.05, 1.08)	<0.001	1.08 (1.06, 1.09)	<0.001	1.44 (1.34, 1.55)	<0.001
Low[a]	1 [Reference]		1 [Reference]		1 [Reference]	
High[a]	1.35 (1.27, 1.44)	<0.001	1.45 (1.34, 1.56)	<0.001	1.44 (1.33, 1.55)	<0.001
T1[b]	1 [Reference]		1 [Reference]		1 [Reference]	
T2[b]	1.16 (1.06, 1.27)	0.001	1.17 (1.06, 1.3)	0.002	1.16 (1.05, 1.29)	0.004
T3[b]	1.48 (1.35, 1.62)	<0.001	1.58 (1.42, 1.77)	<0.001	1.57 (1.4, 1.75)	<0.001
P for trend		<0.001		<0.001		<0.001

Model 0 was not adjusted for any covariates.

Model 1 was adjusted for sex, age, TNM, cancer types.

Model 2 was adjusted for sex, age, TNM, cancer types, surgery, chemotherapy, radiotherapy, occupation, BMI.

Low[a]:<22; High[a]:≥22.

T1[b]:<20; T2[b]:20-32; T3[b]:≥32.

Table S12 : Sensitivity Analysis of the Association Between Smoking Duration and All-Cause Mortality in Malnutrition Patients.

Excluding patients with follow-up less than 1 year	HR (95% CI)	P value	HR (95% CI)	P value	HR (95% CI)	P value
As continuous （per 10 years)	1.1 (1.08, 1.12)	<0.001	1.06 (1.03, 1.08)	<0.001	1.72 (1.41, 2.11)	<0.001
Low[a]	1 [Reference]		1 [Reference]		1 [Reference]	
High[a]	1.39 (1.32, 1.47)	<0.001	1.19 (1.11, 1.28)	<0.001	1.19 (1.11, 1.28)	<0.001
T1[b]	1 [Reference]		1 [Reference]		1 [Reference]	
T2[b]	1.14 (1.05, 1.23)	0.002	1.06 (0.95, 1.17)	0.302	1.06 (0.95, 1.17)	0.287
T3[b]	1.43 (1.35, 1.51)	<0.001	1.22 (1.12, 1.33)	<0.001	1.22 (1.13, 1.33)	<0.001
P for trend		<0.001		<0.001		<0.001
Excluding patients with BMI>35kg/m^2						

As continuous （per 10 years)	1.11 (1.1, 1.13)	<0.001	1.06 (1.04, 1.08)	<0.001	1.86 (1.55, 2.23)	<0.001
Low[a]	1 [Reference]		1 [Reference]		1 [Reference]	
High[a]	1.46 (1.39, 1.54)	<0.001	1.24 (1.16, 1.32)	<0.001	1.24 (1.16, 1.32)	<0.001
T1[b]	1 [Reference]		1 [Reference]		1 [Reference]	
T2[b]	1.14 (1.06, 1.23)	0.001	1.02 (0.93, 1.12)	0.731	1.02 (0.93, 1.12)	0.673
T3[b]	1.5 (1.42, 1.58)	<0.001	1.25 (1.16, 1.35)	<0.001	1.25 (1.16, 1.35)	<0.001
P for trend		<0.001		<0.001		<0.001
Additionally adjusted for time-varying covariates						
As continuous （per 10 years)	1.11 (1.1, 1.13)	<0.001	1.09 (1.07, 1.11)	<0.001	2.39 (1.99, 2.88)	<0.001
Low[a]	1 [Reference]		1 [Reference]		1 [Reference]	
High[a]	1.46 (1.39, 1.54)	<0.001	1.34 (1.26, 1.44)	<0.001	1.34 (1.25, 1.43)	<0.001
T1[b]	1 [Reference]		1 [Reference]		1 [Reference]	
T2[b]	1.14 (1.06, 1.23)	0.001	1.03 (0.93, 1.13)	0.574	1.04 (0.94, 1.14)	0.448
T3[b]	1.5 (1.42, 1.58)	<0.001	1.36 (1.27, 1.47)	<0.001	1.37 (1.27, 1.47)	<0.001
P for trend		<0.001		<0.001		<0.001

Model 0 was not adjusted for any covariates.

Model 1 was adjusted for sex, age, TNM, cancer types.

Model 2 was adjusted for sex, age, TNM, cancer types, surgery, chemotherapy, radiotherapy, occupation, BMI.

Low[a]:<25; High[a]: ≥25.

T1[b]:0; T2[b]:0-20; T3[b]:≥20.

Figure S12 : Hazard ratios (HR) for all-cause mortality with 95% confidence intervals are shown for smoking in relation to CRP and WBC.

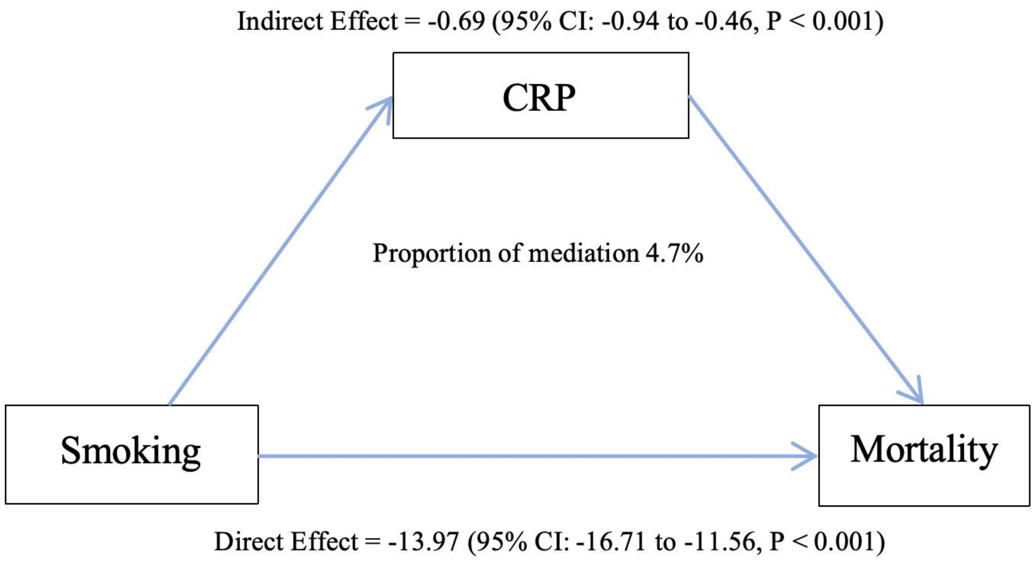

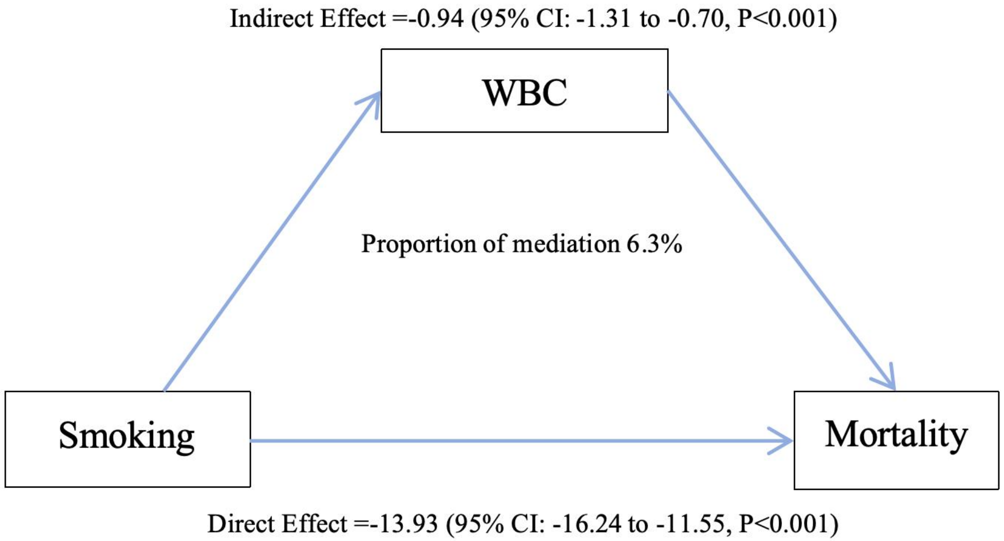
Indirect Effect =-0.94 (95% CI: -1.31 to -0.70, P<0.001)
WBC
Proportion of mediation 6.3%
Smoking
Mortality
Direct Effect =-13.93 (95% CI: -16.24 to -11.55, P<0.001)

Figure S13 : Hazard ratios (HR) for all-cause mortality with 95% confidence intervals are shown for PG-SGA in relation to CRP and WBC.

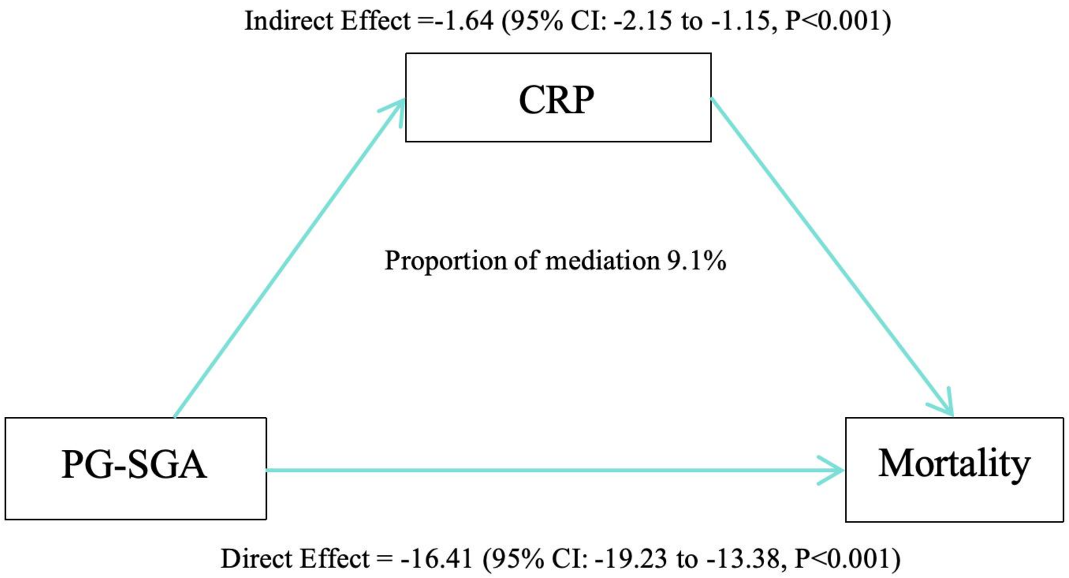

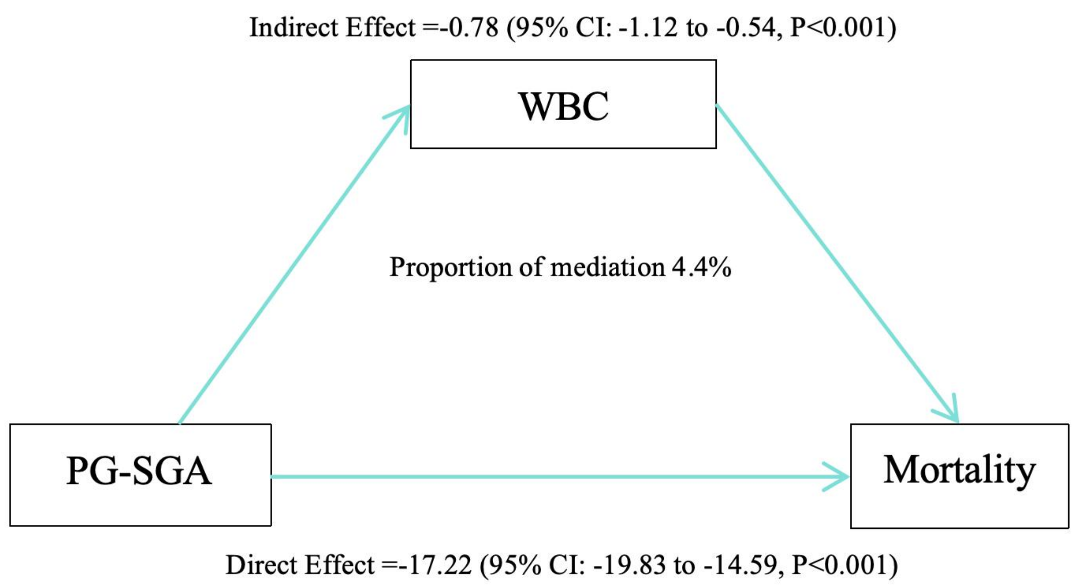
Indirect Effect =-0.78 (95% CI: -1.12 to -0.54, P<0.001)
WBC
Proportion of mediation 4.4%
PG-SGA
Mortality
Direct Effect =-17.22 (95% CI: -19.83 to -14.59, P<0.001)
